# Construction of a circRNA– lincRNA–lncRNA–miRNA–mRNA ceRNA regulatory network identifies genes and pathways linked to goat fertility

**DOI:** 10.3389/fgene.2023.1195480

**Published:** 2023-07-21

**Authors:** Farzad Ghafouri, Mostafa Sadeghi, Abolfazl Bahrami, Masoumeh Naserkheil, Vahid Dehghanian Reyhan, Arash Javanmard, Seyed Reza Miraei-Ashtiani, Soheila Ghahremani, Herman W. Barkema, Rostam Abdollahi-Arpanahi, John P. Kastelic

**Affiliations:** ^1^ Department of Animal Science, College of Agriculture and Natural Resources, University of Tehran, Karaj, Iran; ^2^ Nuclear Agriculture Research School, Nuclear Science and Technology Research Institute, Karaj, Iran; ^3^ Animal Breeding and Genetics Division, National Institute of Animal Science, Cheonan-si, Republic of Korea; ^4^ Department of Animal Sciences, Faculty of Agriculture, University of Tabriz, Tabriz, Iran; ^5^ Department of Animal Science, Faculty of Agriculture, University of Tarbiat Modares, Tehran, Iran; ^6^ Faculty of Veterinary Medicine, University of Calgary, Calgary, AB, Canada

**Keywords:** fertility, ceRNA regulatory network, ovarian granulosa cells, goat, scRNA-seq analysis

## Abstract

**Background:** There is growing interest in the genetic improvement of fertility traits in female goats. With high-throughput genotyping, single-cell RNA sequencing (scRNA-seq) is a powerful tool for measuring gene expression profiles. The primary objective was to investigate comparative transcriptome profiling of granulosa cells (GCs) of high- and low-fertility goats, using scRNA-seq.

**Methods:** Thirty samples from Ji’ning Gray goats (*n* = 15 for high fertility and *n* = 15 for low fertility) were retrieved from publicly available scRNA-seq data. Functional enrichment analysis and a literature mining approach were applied to explore modules and hub genes related to fertility. Then, interactions between types of RNAs identified were predicted, and the ceRNA regulatory network was constructed by integrating these interactions with other gene regulatory networks (GRNs).

**Results and discussion:** Comparative transcriptomics-related analyses identified 150 differentially expressed genes (DEGs) between high- and low-fertility groups, based on the fold change (≥5 and ≤−5) and false discovery rate (FDR <0.05). Among these genes, 80 were upregulated and 70 were downregulated. In addition, 81 mRNAs, 58 circRNAs, 8 lincRNAs, 19 lncRNAs, and 55 miRNAs were identified by literature mining. Furthermore, we identified 18 hub genes (*SMAD1*, *SMAD2*, *SMAD3*, *SMAD4*, *TIMP1*, *ERBB2*, *BMP15*, *TGFB1*, *MAPK3*, *CTNNB1*, *BMPR2*, *AMHR2*, *TGFBR2*, *BMP4*, *ESR1*, *BMPR1B*, *AR*, and *TGFB2*) involved in goat fertility. Identified biological networks and modules were mainly associated with ovary signature pathways. In addition, KEGG enrichment analysis identified regulating pluripotency of stem cells, cytokine–cytokine receptor interactions, ovarian steroidogenesis, oocyte meiosis, progesterone-mediated oocyte maturation, parathyroid and growth hormone synthesis, cortisol synthesis and secretion, and signaling pathways for prolactin, TGF-beta, Hippo, MAPK, PI3K-Akt, and FoxO. Functional annotation of identified DEGs implicated important biological pathways. These findings provided insights into the genetic basis of fertility in female goats and are an impetus to elucidate molecular ceRNA regulatory networks and functions of DEGs underlying ovarian follicular development.

## 1 Introduction

Improvement in reproductive performance is a priority in the goat industry as it is one of the most important determinants of productivity, sustainability, and profitability. The reproductive cycle involves dynamic and complex ovarian functions, characterized by progressive emergence and development of ovarian follicles (Fatet et al., 2011) under endocrine control. Both granulosa and theca cells are involved in steroidogenesis (Qiu et al., 2013). Granulosa cells (GCs) are one of the most important cell types in ovarian follicles, playing crucial roles in follicular development and atresia (Matsuda et al., 2012), especially in the late stages of oocyte development and ovulation. In addition, these cells also control cytoplasmic maturation and play a key role in nuclear maturation by responding to gonadotropins (Mori et al., 2000). Thus, reproductive function in the female is inherently complex, involving various anatomical and physiological processes (Li et al., 2021a). Furthermore, litter size, an important attribute directly related to reproductive efficiency, is controlled by multiple genes and factors (Lai et al., 2016). Hence, knowledge of the genetic basis of reproductive efficiency will provide insights into components controlling ovarian folliculogenesis and fertility in goats (de Lima et al., 2020).

The candidate gene approach for fertility has been extensively studied in various livestock species (Miao et al., 2016a; Miao et al., 2016b; Bahrami et al., 2017; Quan et al., 2019). Furthermore, it requires well-developed tools to detect and characterize multiple genes, pathways, and networks (Ahlawat et al., 2016; Zhang et al., 2017; Ghafouri et al., 2021; Naserkheil et al., 2022).

Single-cell RNA sequencing (scRNA-seq) has been used to characterize transcripts and differences in gene expression, identify functional genes, and analyze regulatory networks in numerous species (Chen et al., 2015; Li et al., 2021b; Li et al., 2021c). In addition, scRNA-seq is being used for mapping and quantifying transcriptional activity at single-cell resolution for all genes in the genome (Islam et al., 2014). It is useful for analysis of cellular heterogeneity as it can concurrently sequence thousands of cells and discover novel cell types in animals (Choi and Kim, 2019). Conversely, an integrated approach is needed to manage large-scale data generated with high-throughput technologies alongside literature mining. Integrated analyses can combine multilevel views of physiology data into a total interpretation of nonlinear regulatory molecular procedures (La et al., 2019; Reyhan et al., 2022; Sadeghi et al., 2022). Currently, various bioinformatics tools, computational approaches, and algorithms are available to identify interactions and protein functions in regulatory modules in various complex biological networks (Bugrim et al., 2004). In this regard, multi-partite networks such as circRNA–lincRNA–lncRNA–miRNA–mRNA ceRNA regulatory networks consider various RNAs and have highlighted a new regulatory mechanism of interaction among RNAs. Circular RNAs (circRNAs) are single-stranded, covalently closed RNA molecules without free 5′ and 3′ ends that exert biological function by acting as transcriptional regulators, microRNA sponges, and protein templates (Zhou et al., 2020). Long intergenic non-coding RNAs (lincRNAs), RNA transcripts with >200 nucleotides, play major roles in biological processes such as gene expression control, epigenetic control, and scaffold formation (Deniz and Erman, 2017). Long non-coding RNAs (lncRNAs) are non-coding RNA transcripts involved in various biological procedures, such as cell proliferation and transcriptional regulation (Wei et al., 2016). In addition, microRNAs (miRNAs), regulatory molecules with 19–25 nucleotides, play vital regulatory roles in multiple biological procedures (cell differentiation and migration, oncogenesis, and apoptosis) by suppressing mRNAs (Wang et al., 2015). Therefore, this approach seems well-suited to understand molecular regulatory mechanisms in polygenic traits (Hallock and Thomas, 2012).

Several studies have identified important candidate genes associated with hormonal regulation of the reproductive cycle and fertility traits in goats (Li et al., 2010; Ahlawat et al., 2016; Lai et al., 2016; Zhang et al., 2018a; Li et al., 2021b). Furthermore, gene ontology and systems biology enable identification of hub genes and co-expression genes with critical roles in fertility (Ahlawat et al., 2016; Zhang et al., 2018b). For instance, in a study comparing high- *versus* low-fertility goats, many candidate genes were identified in each group. In an analysis of the entire genome of Chinese Laoshan dairy goats, several candidate genes (*CCNB2*, *AR*, *SMAD2*, *AMHR2*, *KDM6A*, *SOX5*, and *SYCP2*) were associated with both high and low fertility (Lai et al., 2016). Therefore, the purpose of the present study was to examine the ovarian GC signature genomic regions of Ji’ning Gray goats with high and low fertility, using a public repository scRNA-seq dataset. In addition, we critically reviewed the literature, searching for relevant publications using keywords related to GCs and fertility in goats. Genes with significant differences related to our bioinformatics analyses and candidate gene list extracted from literature mining were compiled, and the two gene sets were merged and used to identify protein–protein interaction (PPI) networks and gene regulatory networks (GRNs). Overall, the ceRNA regulatory network, consisting of circRNA–lincRNA–lncRNA–miRNA–mRNA, was constructed based on various interactions to explore the effects of functional modules and hub differentially expressed mRNAs, miRNAs, lncRNAs, lincRNAs, and circRNAs on fertility.

## 2 Materials and methods

**FIGURE 1:**
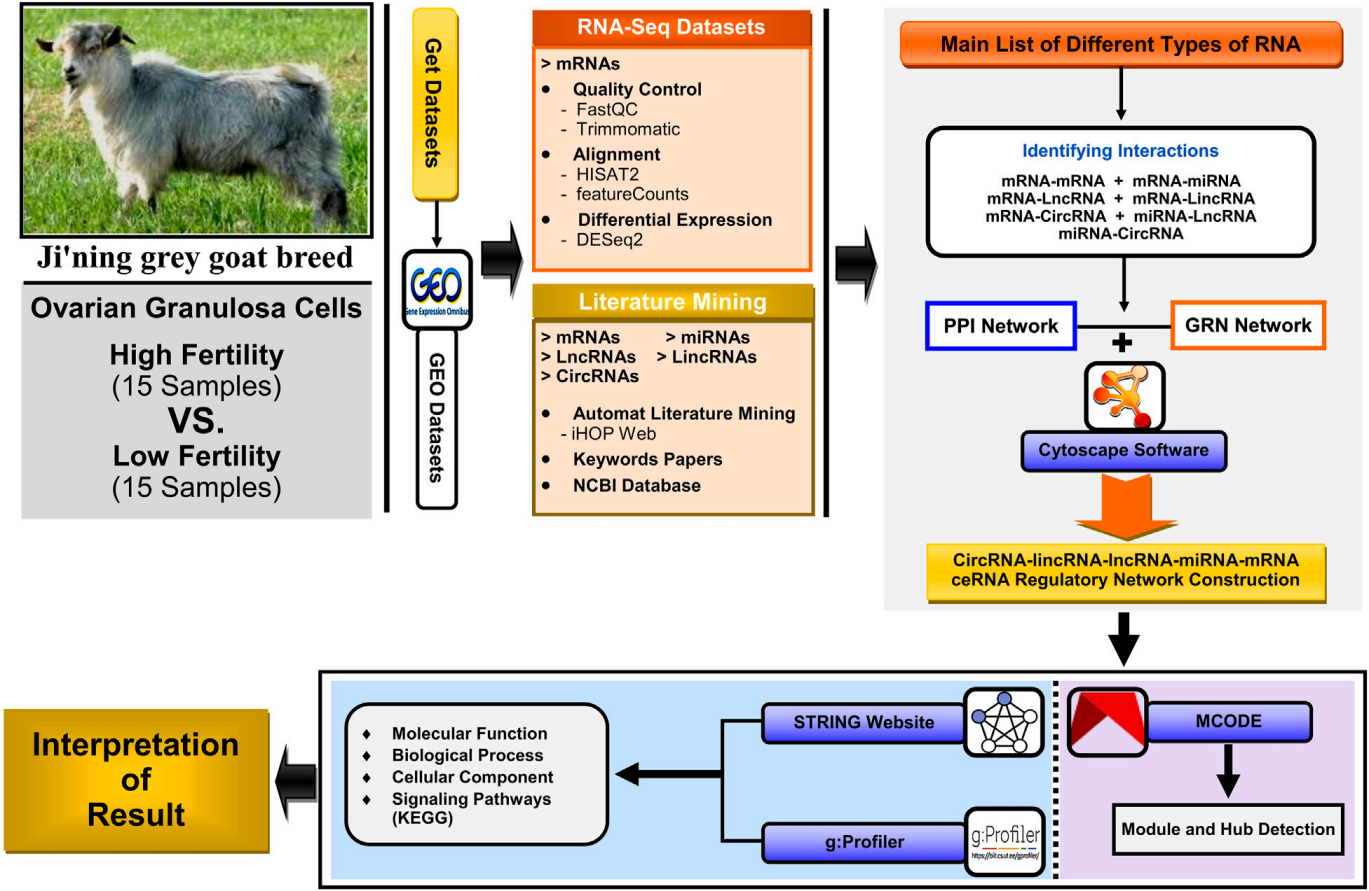
Workflow for analyzing the scRNA-seq dataset, literature mining, protein–protein interaction (PPI) network, gene regulatory network (GRN), and downstream ontology functions, constructing the circRNA–lincRNA–lncRNA–miRNA–mRNA ceRNA regulatory network; module analyses were constructed and visualized using Cytoscape software v3.9.1.

As an overview, the general workflow for analyzing data collection and methods of identifying key genes, metabolic and signaling pathways, and construction of the lncRNA–miRNA–mRNA ceRNA regulatory network and modules affecting high fertility (HF) and low fertility (LF) in domestic goats (*Capra hircus*) is shown in Figure 1.

### 2.1 Data collection

scRNA-seq data from ovarian GCs of high- and low-fertility Ji’ning Gray goats (HF and LF, respectively) were retrieved from the National Center for Biotechnology Information (NCBI) Gene Expression Omnibus (GEO) public database under the accession number GSE135897 (www.ncbi.nlm.nih.gov/geo). This dataset was produced using the GPL15473 Illumina HiSeq 2000 platform (Li et al., 2021b). A case–control study approach was designed to identify differentially expressed genes (DEGs) between HF and LF goats. A total of 30 samples from Ji’ning Gray goats (first group: 15 with high litter size (HF; ≥3 offspring) and second group: 15 with low litter size (LF; ≤2 offspring)) were analyzed. The Ji’ning Gray goat is a local breed and the oldest domesticated goat species with high fertility in China that has year-round estrus and a mean litter size of 2.94 (Huang et al., 2012; Miao et al., 2016a; b). All ovarian follicles from each group of these Ji’ning Gray goats (from small (<3 mm) to large (˃7 mm)) were collected. Then, oocytes and GCs were mechanically separated by repeated pipetting. Oocytes from high- and low-fertility groups were labeled. This study was performed in accordance with ARRIVE guidelines. Library preparation and sequencing were performed separately for oocytes and GCs. Details regarding animal ethics approval, total RNA extraction, separate library preparation, sequencing, and validation of scRNA-seq data have been reported (Zhao et al., 2020; Li et al., 2021b).

### 2.2 Quality control and detection of differentially expressed genes

First, the quality of the raw RNA sequences was assessed using FastQC software v0.11.5 (Andrew, 2010) and FastQ Groomer software v1.1.5 (Blankenberg et al., 2010); thereafter, these sequences were pre-processed with Trimmomatic software v0.38.0 (Bolger et al., 2014) to remove adapters, low-quality reads, and PCR primers. Alignment sequences, mapping, and identification of known and novel RNAs of reads were related to the reference genome of *C. hircus* (https://ftp.ensembl.org/pub/release-108/fasta/capra_hircus/dna/) using HISAT2 software v2.2.1 with default parameters to determine the number of aligned and unaligned reads (Kim et al., 2015). Regarding transcript quacation, total raw counts of mapped reads were calculated using featureCounts software (v2.0.1) (Liao et al., 2014). Subsequently, to examine whether accumulation or degradation of transcripts was related to fertility, transcripts and their expression levels were compared between ovarian GC samples of high- and low-fertility Ji’ning Gray goats. Differences in gene expression were detected from reading counts using DESeq2 software (v2.11.40.7) (Love et al., 2014). The threshold for statistical significance of the differential expression of each gene was obtained with the criteria of log2FC (fold change ≥5 and ≤−5) and FDR <0.05 (false discovery rate).

### 2.3 Literature mining to identify candidate genes for fertility in goats

Various online databases were examined to discover candidate circRNAs, lincRNAs, lncRNAs, miRNAs, and mRNAs relevant to comprehensive literature mining. Online search databases and papers included Google Scholar, PubMed, Web of Science, iHOP web services, and CrossRef from 2010 to 2023, with no language restrictions. Search terms consisted of both keywords and database-specific subject headings for the ceRNA regulatory network, GCs, and fertility in goats: breeds–goats; practical tools–scRNA-seq; and outcome–ceRNA network or regulatory RNAs-fertility, and litter size trait. Keywords included goats, fertility, litter size, ovarian GCs, circRNA, lincRNA, lncRNA, miRNA, mRNA, and ceRNA networks. For this purpose, first, identifiers and synonyms for each framework element were merged by applying the Boolean operator “OR.” Then, elements of the framework were merged by applying the Boolean operator “AND.” In total, 74 relevant papers were identified using online search databases. All identified papers were imported into Covidence (Covidence systematic review software, Veritas Health Innovation), and duplicates were removed. The included articles were further screened for relevant references, and a citation check was performed. After the final screening, 41 papers with the final qualified literature were listed, and regulatory RNAs (i.e., circRNAs, lincRNAs, lncRNAs, miRNAs, and mRNAs) with significant differences related to the candidate RNA list extracted from literature mining were compiled as RNA sets 2–6, respectively (Supplementary Tables S2–6). Finally, RNA set 1 (from our bioinformatics analyses) and RNA sets 2–6 (from literature mining) were merged and used as input files for target prediction tools, the STRING website, and Cytoscape software to identify PPI GRNs, and reconstruct the circRNA–lincRNA–lncRNA–miRNA–mRNA ceRNA regulatory network and modules.

### 2.4 Gene ontology and functional enrichment analysis

Gene set annotation and enrichment analysis used DAVID (the Database for Annotation, Visualization, and Integrated Discovery; https://david.ncifcrf.gov/) v6.8 (Sherman and Lempicki, 2009), g:Profiler (https://biit.cs.ut.ee/gprofiler/gost) (Reimand et al., 2016), GeneCards (https://www.genecards.org/), and STRING database v11.0 (https://string-db.org) (Szklarczyk et al., 2019) to determine potential functions and metabolic pathways. Genes were assigned to functional categories using the Gene Ontology (GO) database under biological process (BP), molecular function (MF), and cellular component (CC).

### 2.5 Target prediction of differentially expressed mRNAs and other types of regulatory RNAs

Functional annotation of types of regulatory RNAs, i.e., circRNAs, lincRNAs, lncRNAs, and miRNAs, consisted of functional annotation of their potential target mRNA genes. Predicted targeted genes and types of regulatory RNAs were predicted using miRBase (Kozomara et al., 2019) (https://www.mirbase.org/), TargetScan (Grimson et al., 2007), miRanda (http://www.microrna.org/), miRWalk 3.0 (a comprehensive atlas of microRNA–target interaction tools that integrates 12 miRNA–target prediction tools; http://mirwalk.umm.uni-heidelberg.de/), NONCODE database (Volders et al., 2019) (http://www.noncode.org/), LNCipedia database (Bader et al., 2006) (https://lncipedia.org), and CircInteractome web tool (Dudekula et al., 2016) (a computational tool that enables prediction and mapping of binding sites for RBPs and miRNAs on reported circRNAs; https://circinteractome.nia.nih.gov/). Identified target genes were selected and submitted to DAVID, KEGG, Reactome pathways, and the PANTHER database for enrichment and validation of target genes for each type of RNA.

### 2.6 CircRNA–lincRNA–lncRNA–miRNA–mRNA ceRNA regulatory network construction

Based on the ceRNA theory, global functions for all non-coding RNAs can serve as an endogenous “sponge” to regulate upregulated or downregulated circRNAs, lncRNAs, lincRNAs, miRNAs, or mRNAs that have inverse relationships together in the mRNA–mRNA, mRNA–miRNA, mRNA–lncRNA, mRNA–lincRNA, mRNA–circRNA, miRNA–lncRNA, and miRNA–circRNA interaction pairs chosen to construct the circRNA–lincRNA–lncRNA–miRNA–mRNA ceRNA regulatory network (Xu et al., 2019). In this regard, PPI network analysis was performed using the STRING database v11.0 (Szklarczyk et al., 2019) (Search Tool for the Retrieval of Interacting Genes or Proteins; https://string-db.org), BIND (Biomolecular Interaction Network Database) (Bader et al., 2003), MIPS (Mammalian Protein–Protein Interaction Database) (Pagel et al., 2005), and BioGRID (Biological General Repository for Interaction Datasets) (Chatr-Aryamontri et al., 2012) to explore interactions between genes in *C. hircus*. After identifying interactions between types of regulatory RNAs and gene expression data (co-expression), the circRNA–lincRNA–lncRNA–miRNA–mRNA ceRNA regulatory network was reconstructed and plotted using Cytoscape software v3.9.1. (Shannon et al., 2003; National Institute of General Medical Sciences, Bethesda Softworks, Rockville, MD, USA). Furthermore, statistical and topological significance of the network was assessed with the Network Analyzer plugin in Cytoscape software.

### 2.7 Clustering of the circRNA–lincRNA–lncRNA–miRNA–mRNA ceRNA regulatory network and identification of main hub regulatory RNAs

Modules or subnets may play a significant role in the biologically rebuilt main ceRNA regulatory network as they represent a set of nodes with similar functions that pursue specific biological purposes as functional modules. To evaluate topological properties and cluster nodes of the circRNA–lincRNA–lncRNA–miRNA–mRNA ceRNA regulatory network, the Cytoscape plugins ClusterONE (Shannon et al., 2003) and MCODE (Lotia et al., 2013), clustering algorithms to draw directional and directionless graphs, were used. ClusterONE is a plugin to discover densely connected sub-graphs of a network by minimizing edges between clusters and maximizing edges within a cluster. In addition, the MCODE plugin can be used for directed or undirected graphs. Various parameters and the statistical and topological significance of the ceRNA regulatory modules were calculated using the Cytoscape plugin Network Analyzer v4.4.8 with the default for the directed network (Assenov et al., 2008).

## 3 Results and discussion

### 3.1 scRNA-seq analysis to identify differentially expressed genes

In this study, we investigated the pattern of transcriptome profiles of ovarian GCs in goats. To provide insights into the genetic basis of fertility in Ji’ning Gray goats, gene expression analyses using the scRNA-seq dataset with the access number GSE135897 (obtained from the GEO database) were used. The selected gene expression profile had 30 samples, including 15 samples of GCs from high-fertility goats and 15 samples of GCs from low-fertility goats. To perform this analysis, these 30 samples were divided into two groups (high- and low-fertility) to compare expressions of gene profiles and identify significant DEGs. A total of 3,245 significant genes were identified by processing the expression profile of GCs of high- *versus* low-fertility goats. Finally, by considering the expression change threshold (fold change ≥5 and ≤−5, FDR <0.05), 150 genes were significantly differentially expressed in GCs from goats with high *versus* low fertility. Of these genes, 80 genes were upregulated and 70 were downregulated (Supplementary Table S1).

Recently, many studies in molecular genetics, bioinformatics, and biological systems have been conducted to discover candidate genes and identify molecular pathways involved in fertility (Ahlawat et al., 2020; An et al., 2021; Li et al., 2021b). The most identified genes were related to reproductive functions and strongly regulated ovarian follicular growth and secretion of hormones involved in fertility; consequently, increasing or decreasing their expression at various times can lead to complex BPs during pregnancy (Wang et al., 2019). In this regard, in a study using scRNA-seq analysis for ovarian tissue of pregnant and non-pregnant goats, four genes (*PGR*, *PRLR*, *STAR*, and *CYP19A1*) were identified to play important roles in goat reproduction (Quan et al., 2019). In addition, another study concluded that some lncRNAs in goats play key roles in regulating follicle development and cell growth during ovarian development (Li et al., 2021c).

### 3.2 Literature mining-based evidence for identified DEGs and types of regulatory RNAs

Literature mining provided evidence for 81 well-known DEGs that were not included in our investigated outputs; adding this list of DEGs to our discovered DEGs created a good platform for further downstream analyses, including GO and functional enrichment analysis. Identified DEGs based on literature mining and their role in fertility in goats were collected as RNA set 2 (Supplementary Table S2). In addition, literature mining provided evidence for 58, 8, 19, and 55 well-known types of regulatory RNAs, i.e., circRNAs, lincRNAs, lncRNAs, and miRNAs with significant differences, compiled as RNA sets 3–6, respectively (Supplementary Tables S3–6). The gene list in Supplementary Table S2 was merged with the gene list in Supplementary Table S1 and used to identify intergenic interactions and reconstruct the PPI network. Then, after identifying interactions between types of regulatory RNAs in RNA sets 3–6 together and with gene expression data, the circRNA–lincRNA–lncRNA–miRNA–mRNA ceRNA regulatory network was reconstructed.

### 3.3 Gene ontology and pathway enrichment analysis

**FIGURE 2:**
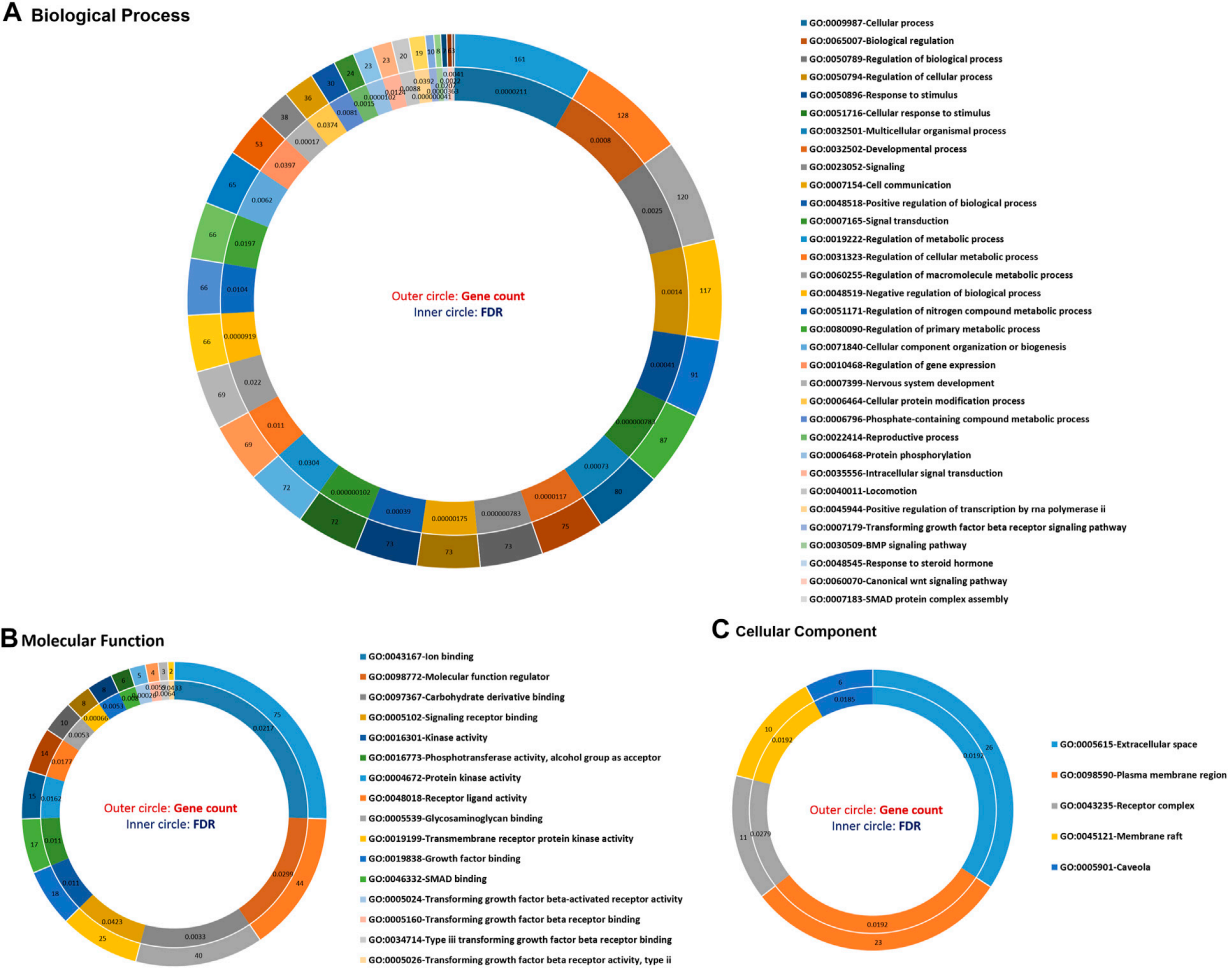
Top significant gene ontology (GO) terms enriched using differentially expressed genes associated with fertility in female goats. **(A)** The significant biological processes, **(B)** the significant molecular function, and **(C)** the significant cellular component GO terms associated with fertility in female goats.

**FIGURE 3:**
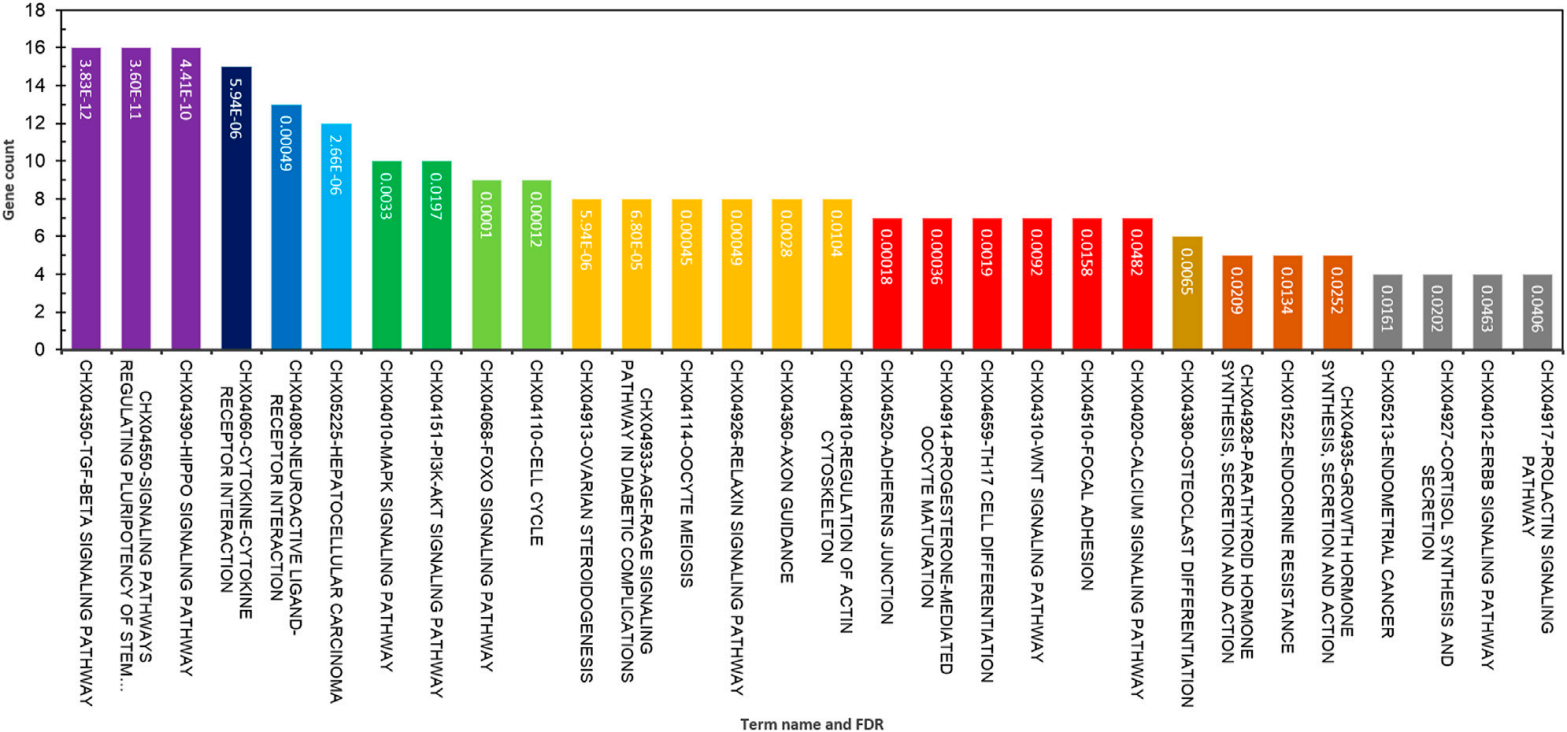
Top significant KEGG pathway terms enriched using differentially expressed genes associated with fertility in female goats.

The functional annotation of GO terms was detected based on BP, MF, and CC to identify functions and metabolic pathways, as well as systematic features of the merged list of 231 DE genes (RNA sets 1 and 2), using the STRING, DAVID, PANTHER, and g:Profiler databases. The results of the GO classification of the DEGs for high- and low-fertility goats are presented in Figure 2. Identified DEGs were significantly involved in the following functions: >10 genes from DEGs played roles in the cellular process, regulation of BP, response to stimulus, cellular response to stimulus, cell communication, regulation of the cellular metabolic process, CC organization or biogenesis, reproductive process, intracellular signal transduction, transforming growth factor beta receptor signaling pathway, BMP signaling pathway, response to steroid hormone, and SMAD protein complex assembly for BPs (Figure 2A). The gene list, including genes *SMAD2*, *ESR1*, *SOX5*, *BMP4*, *BMP15*, *CTNNB1*, *ERBB2*, *FGFR1*, *CDH26*, *GH*, *AR*, and *FSHB*, was involved in most of the MF terms that can be considered significant genes. Moreover, 16 MF terms were identified, such as ion binding, transmembrane receptor protein kinase activity, signaling receptor binding, MF regulator, receptor–ligand activity, growth factor binding, and transforming growth factor beta-activated receptor activity, which were the most significant functions associated with fertility (Figure 2B). Regarding CCs, five GO terms, namely, the extracellular space, plasma membrane region, receptor complex, membrane raft, and caveola, were identified (Figure 2C). In addition, KEGG-based pathway analysis for identified DEGs was performed using three online databases, DAVID, STRING, and g:Profiler. The identified DEGs involved in fertility were enriched in the signaling pathways regulating pluripotency of stem cells, cytokine–cytokine receptor interaction, ovarian steroidogenesis, oocyte meiosis, progesterone-mediated oocyte maturation, parathyroid hormone synthesis, secretion and action, growth hormone synthesis, secretion and action, cortisol synthesis and secretion, prolactin, TGF-beta, Hippo, MAPK, PI3K-Akt, and FoxO signaling pathways (Figure 3).

Concerning female reproductive function, *CTNNB1*, *BMP4*, *FSHR*, *TGFB1*, *BMPR1B*, and *ESR1* genes are jointly encoded in functions of the ovulatory cycle process, ovarian follicular development, developmental process involved in reproduction, and cell differentiation. Hence, according to genes involved in the identified pathways, *ESR1* and *BMPR1B* were considered candidate genes for reproductive functions, growth, and cell differentiation in goats. However, many studies have implicated the *BMPR1B* gene as one of the main genes controlling reproductive function and fertility (e.g., ovulation rate) in small ruminants, especially goats (Pramod et al., 2013; Ahlawat et al., 2014). In addition, the *FSHR* (follicle-stimulating hormone receptor) gene plays roles in growth, differentiation, and maturation of follicles and enhances reproductive function in goats and sheep (Chen et al., 2017). Moreover, another study demonstrated that *FSHR* is involved in differential expression of ovarian mRNA hormone receptor genes in goat fertility (Saraiva et al., 2011). The *ESR1* gene encodes estrogen receptor and ligand-activated transcription factor, and regulates the main genes involved in growth, metabolism, and pregnancy. It has also been reported that expression of this gene was highest in the kidney, ovary, uterus, and testes but lowest in brain and heart tissue (Mohammadabadi, 2020). Given the role of the *BMP4* gene in reproduction, especially in growth and differentiation of ovarian follicles and ovulation, it can be considered a main candidate gene for reproductive function and fertility (Sharma et al., 2013). The *CTNNB1* gene encodes a complex of proteins that constitutes adherens junctions (AJs); they are necessary for formation and maintenance of epithelial cell layers by regulating cell growth and intercellular connectivity. In addition, they play an essential role in reproduction and multiplication (Zhang et al., 2018b). Therefore, our findings regarding genes *FSHR*, *CTNNB1*, *BMPR1B*, and *ESR1* were consistent with other studies.

### 3.4 Construction and clustering of the circRNA–lincRNA–lncRNA–miRNA–mRNA ceRNA regulatory network

**FIGURE 4:**
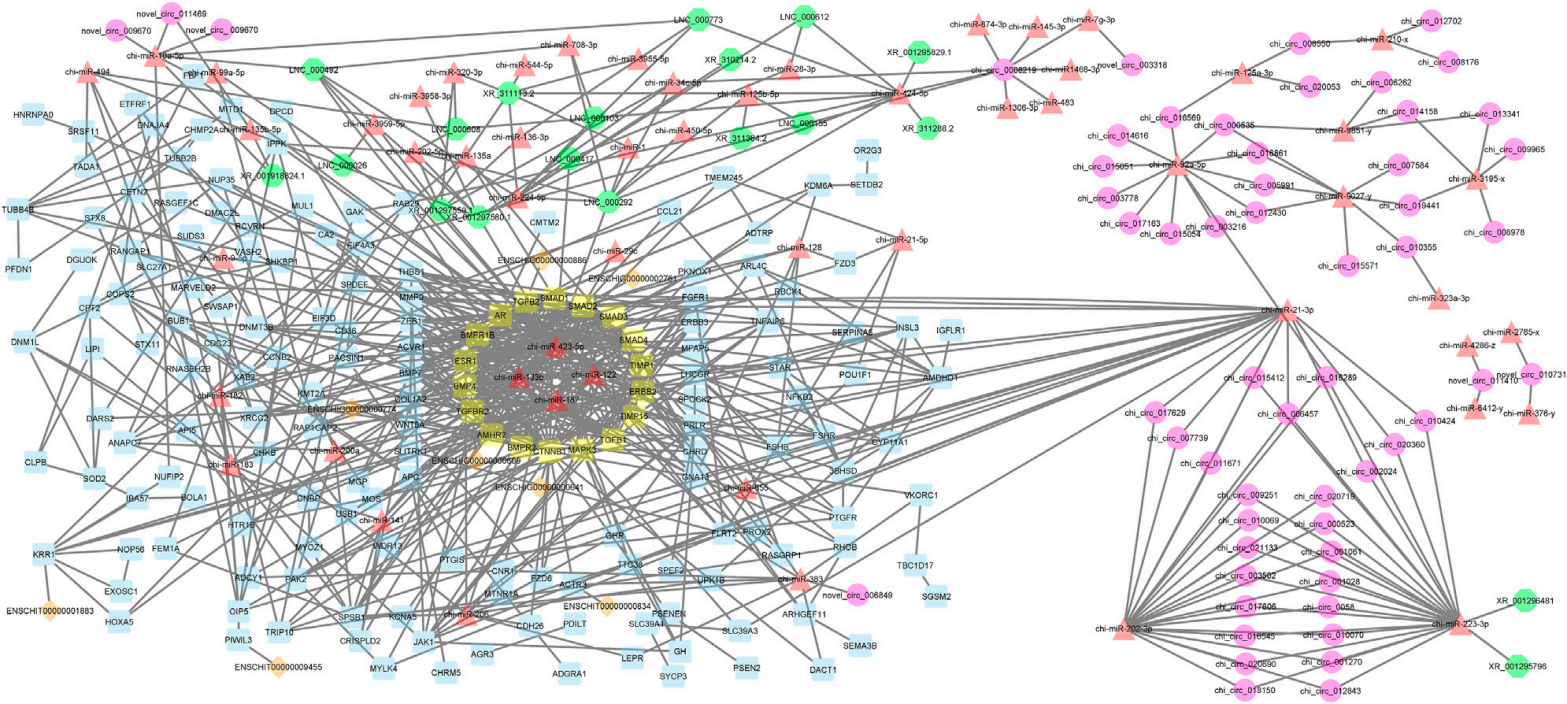
ceRNA regulatory network: 57 circRNAs, 8 lincRNAs, 19 lncRNAs, 51 miRNAs, and 175 mRNAs in an interacted network were identified. In this network, circular nodes represent circRNAs, diamond nodes represent lincRNAs, octagonal nodes represent lncRNAs, triangle nodes represent miRNAs, and quadrilateral nodes represent mRNAs/genes. Yellow quadrilateral nodes represent hub mRNAs/genes involved in the network. Black edges indicate interactions between nodes.

A decade ago, Salmena et al. presented the competitive endogenous RNA hypothesis (Salmena et al., 2011). The ceRNA regulatory networks have provided a new mechanism of interaction among RNAs and play crucial roles in multiple BPs (Kfir et al., 2018; Yang et al., 2019; Gao et al., 2021). In this regard, many studies have been dedicated to elucidating the ceRNA roles of non-coding RNAs in some economically important traits by constructing competitive endogenous RNA networks (Salmena et al., 2011; Han et al., 2020). To detect the mechanism of how non-coding RNAs (ncRNAs) regulate mRNA through sponging miRNA, a ceRNA regulatory network was constructed with a merge of predicted mRNA–miRNA, mRNA–lncRNA, mRNA–lincRNA, mRNA–circRNA, miRNA–lncRNA, and miRNA–circRNA interaction pairs. The reconstructed ceRNA regulatory network for up- and downregulated mRNAs/genes and types of regulatory RNAs, indicating physical connections between two or more protein molecules related to biochemical functions, is presented in Figure 4. Based on knowledge of interactions, this ceRNA regulatory network consisted of 310 nodes and 758 edges and the associated files with the networks are given in Supplementary Table S7 (stored in “.cys format” for further analyses). In detail, 57 circRNAs, 8 lincRNAs, 19 lncRNAs, 51 miRNAs, and 175 mRNAs were included in the network (Figure 4). As mentioned, molecular species (circRNAs, lincRNAs, lncRNAs, mRNAs, and miRNAs) in constructed networks are indicated as nodes and interactions between them as edges. Moreover, constructed networks were combined in a simple interaction format (SIF) using Cytoscape (v3.9.1.) for topological analysis. Topological parameters of the ceRNA regulatory network and modules such as the number of nodes, number of edges, clustering coefficient, characteristic path length, and network density were evaluated to examine the state of communication and information transfer of a node with other nodes of interactive networks, as presented in Table 1. In this ceRNA regulatory network, 18 genes (*SMAD1*, *SMAD2*, *SMAD3*, *SMAD4*, *TIMP1*, *ERBB2*, *BMP15*, *TGFB1*, *MAPK3*, *CTNNB1*, *BMPR2*, *AMHR2*, *TGFBR2*, *BMP4*, *ESR1*, *BMPR1B*, *AR*, and *TGFB2*) had the most interactions with other genes in the network. Among these 18 hub genes, four genes (*BMP4*, *BMPR1B*, *CTNNB1*, and *ESR1*) were involved in MF and BPs, in agreement with Pramod et al. (2013), Sharma et al. (2013), Zhang et al. (2018a), and Mohammadabadi (2020). Among the miRNAs, chi-miR-423-5p, chi-miR-122, chi-miR-187, and chi-miR-133b suppressed most of the identified hub genes as targets of the selected miRNAs. In addition, four of the lincRNAs, i.e., ENSCHIG00000000609, ENSCHIG00000000641, ENSCHIG 00000000886, and ENSCHIG00000002761, interacted with *TGBFR2*, *CTNNB1*, *TGFB2*, and *SMAD2* hub genes, respectively. Conversely, the miRNAs such as chi-miR-92a-5p, chi-miR-21-3p, chi-miR-202-3p, and chi-miR-223-3p interacted with most of the lncRNAs involved in the ceRNA regulatory network and were defined as hub miRNAs that interacted with lncRNAs.

**TABLE 1:** Basic network statistics of the generated main ceRNA regulatory network and its modules.

	Main ceRNA regulatory network	Module			
		1	2	3	4
Number of nodes	310	70	42	77	44
Number of edges	758	282	85	112	59
Clustering coefficient	0.048	0.163	0.112	0.067	0.074
Characteristic path length	2.707	2.019	1.926	1.780	1.736
Network density	0.008	0.058	0.049	0.019	0.031

In this study, clustering of the circRNA–lincRNA –lncRNA–miRNA–mRNA ceRNA regulatory network was performed using ClusterONE (Shannon et al., 2003) and MCODE plugins (Lotia et al., 2013), in accordance with the clustering algorithms utilized to determine significant sub-networks or modules by an integrated approach. There were four candidate modules involved in goat fertility; the node interactions of each component are described in Supplementary Table S7.

**FIGURE 5:**
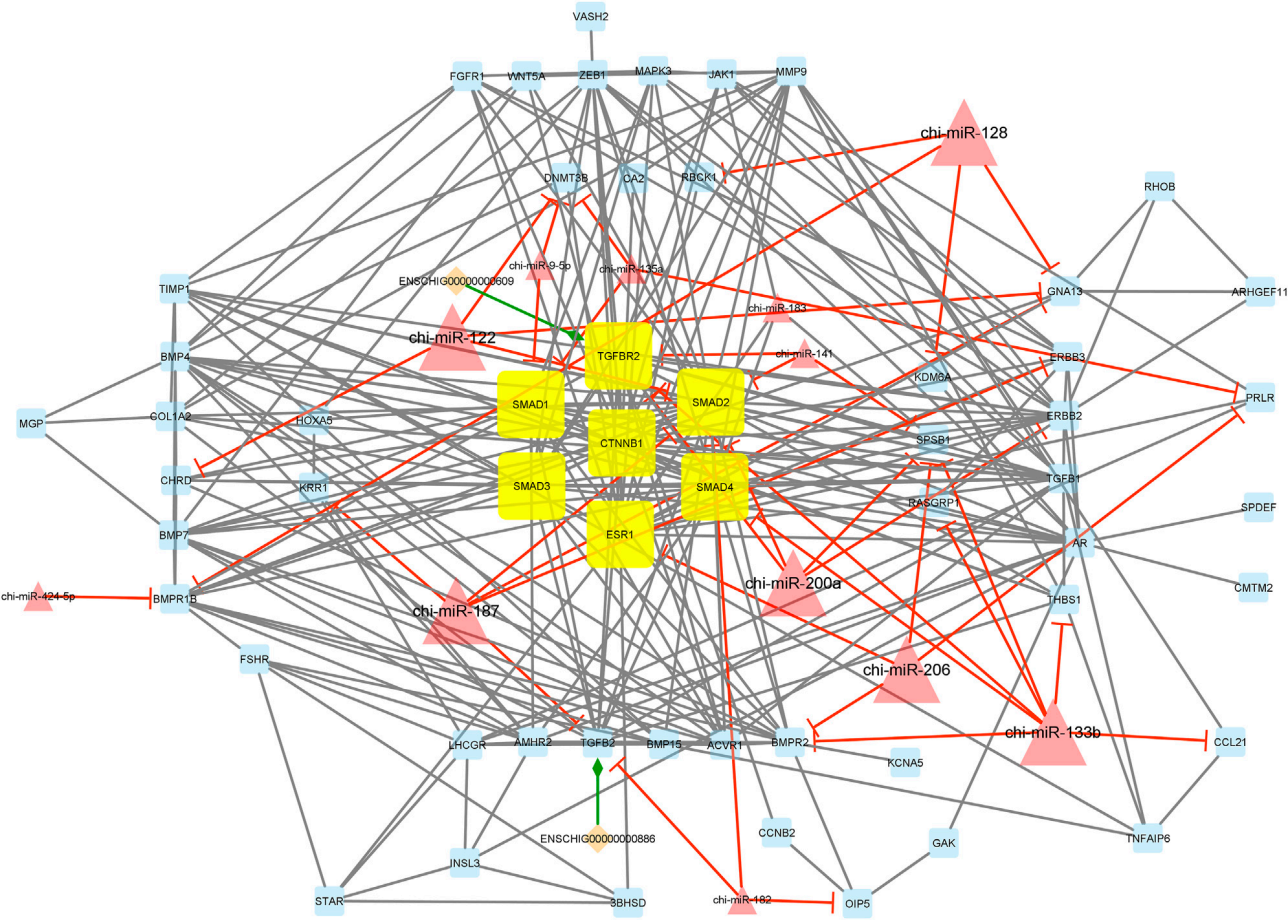
Module 1: 2 lincRNAs, 12 miRNAs, and 56 mRNAs in an interacted network were identified. In this network, diamond nodes represent lincRNAs, triangle nodes represent miRNAs, and quadrilateral nodes represent mRNAs/genes. Yellow quadrilateral and big triangle nodes represent hub mRNAs/genes and miRNAs involved in the network, respectively. Edges indicate the interactions; black edges represent mRNA–mRNA interactions, green edges represent lincRNA–mRNA interactions, and red edges represent miRNA–mRNA interactions.

Interestingly, module 1 consisted of 70 nodes and 282 edges and, in detail, comprised 2 lincRNAs, 12 miRNAs, and 56 mRNAs. In this module, *SMAD1*, *SMAD2*, *SMAD3*, *SMAD4*, *TGFBR2*, *CTNNB1*, and *ESR1* were hub genes. In addition, chi-miR-128, chi-miR-122, chi-miR-187, chi-miR-200a, chi-miR-206, and chi-miR-133b, as hub miRNAs, suppressed most of the involved genes such as *DNMT3B*, *SMAD2*, *SMAD4*, *CHRD*, *RBCK1*, *BMPR1B*, *KDM6A*, *KRR1*, *GNA13*, *ERBB3*, *TGFB2*, *ERBB2*, *SPSB1*, *TGFBR2*, *BMPR2*, *CCL21*, *THBS1*, *PRLR*, *ESR1*, and *RASGRP1*. Moreover, in this module, ENSCHIG00000000609 and ENSCHIG00000000886 lincRNAs interacted with *TGFBR2*, and *TGFB2*, respectively. All hub–hub genes in this module were involved in the metabolic signaling pathways that were analyzed (Figure 5).

**FIGURE 6:**
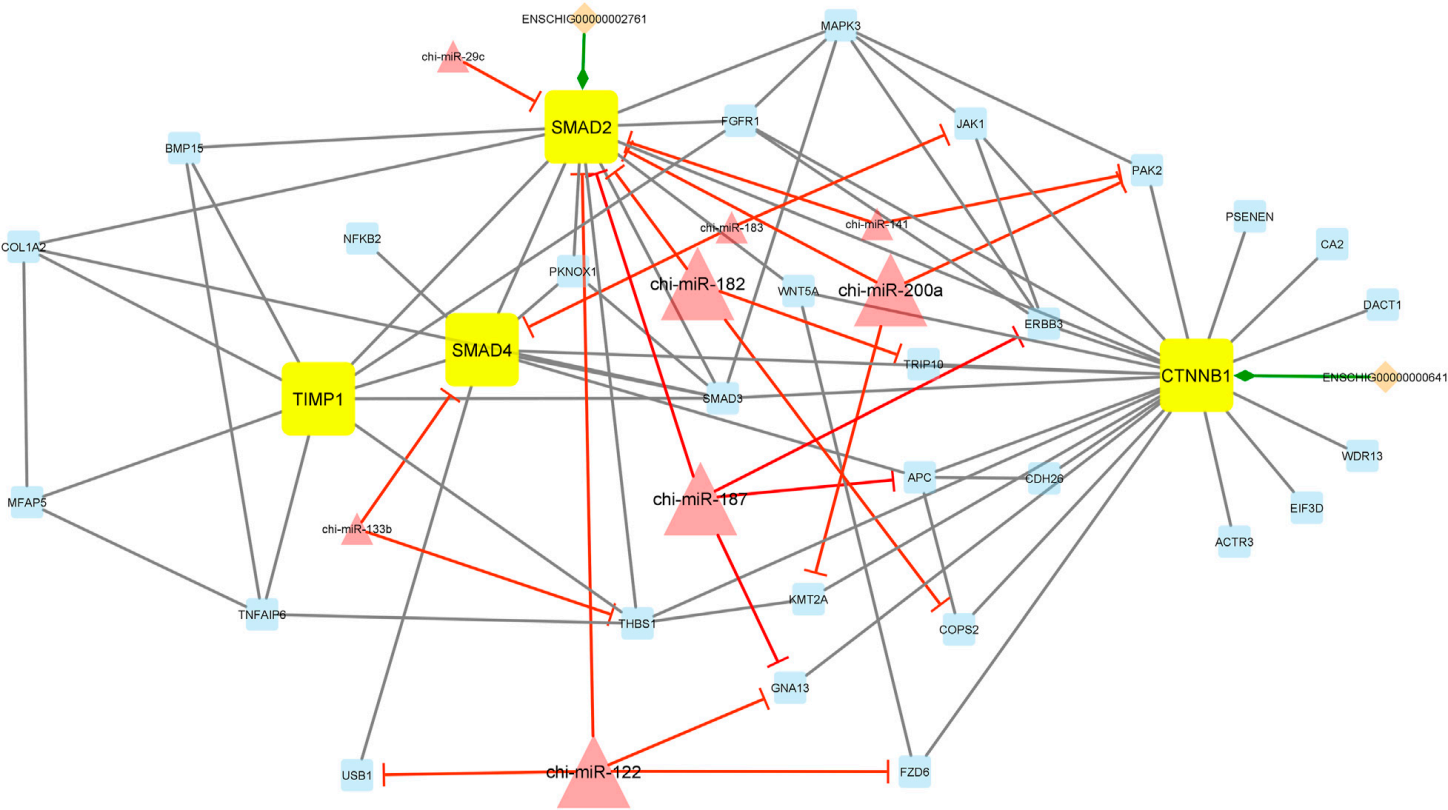
Module 2: 2 lincRNAs, 8 miRNAs, and 32 mRNAs in an interacted network were identified. In this network, diamond nodes represent lincRNAs, triangle nodes represent miRNAs, and quadrilateral nodes represent mRNAs/genes. Yellow quadrilateral and big triangle nodes represent hub mRNAs/genes and miRNAs involved in the network, respectively. Edges indicate the interactions; black edges represent mRNA–mRNA interactions, green edges represent lincRNA–mRNA interactions, and red edges represent miRNA–mRNA interactions.

Additionally, module 2 consisted of 42 nodes and 85 edges and, in detail, comprised 2 lincRNAs, 8 miRNAs, and 32 mRNAs. In this module, *SMAD2*, *SMAD4*, *CTNNB1*, and *TIMP1* were hub genes. In addition, chi-miR-182, chi-miR-200a, chi-miR-187, and chi-miR-122, as hub miRNAs, suppressed most of the genes such as *SMAD2*, *TRIP10*, *COPS2*, *PAK2*, *KMT2A*, *ERBB2*, *APC*, *GNA13*, *USB1*, and *FZD6*. Among these, the *SMAD2* gene was suppressed more than other genes involved in the network. Moreover, in this module, ENSCHIG00000000641 and ENSCHIG00000002761 lincRNAs interacted with *CTNNB1* and *SMAD2*, respectively (Figure 6).

**FIGURE 7:**
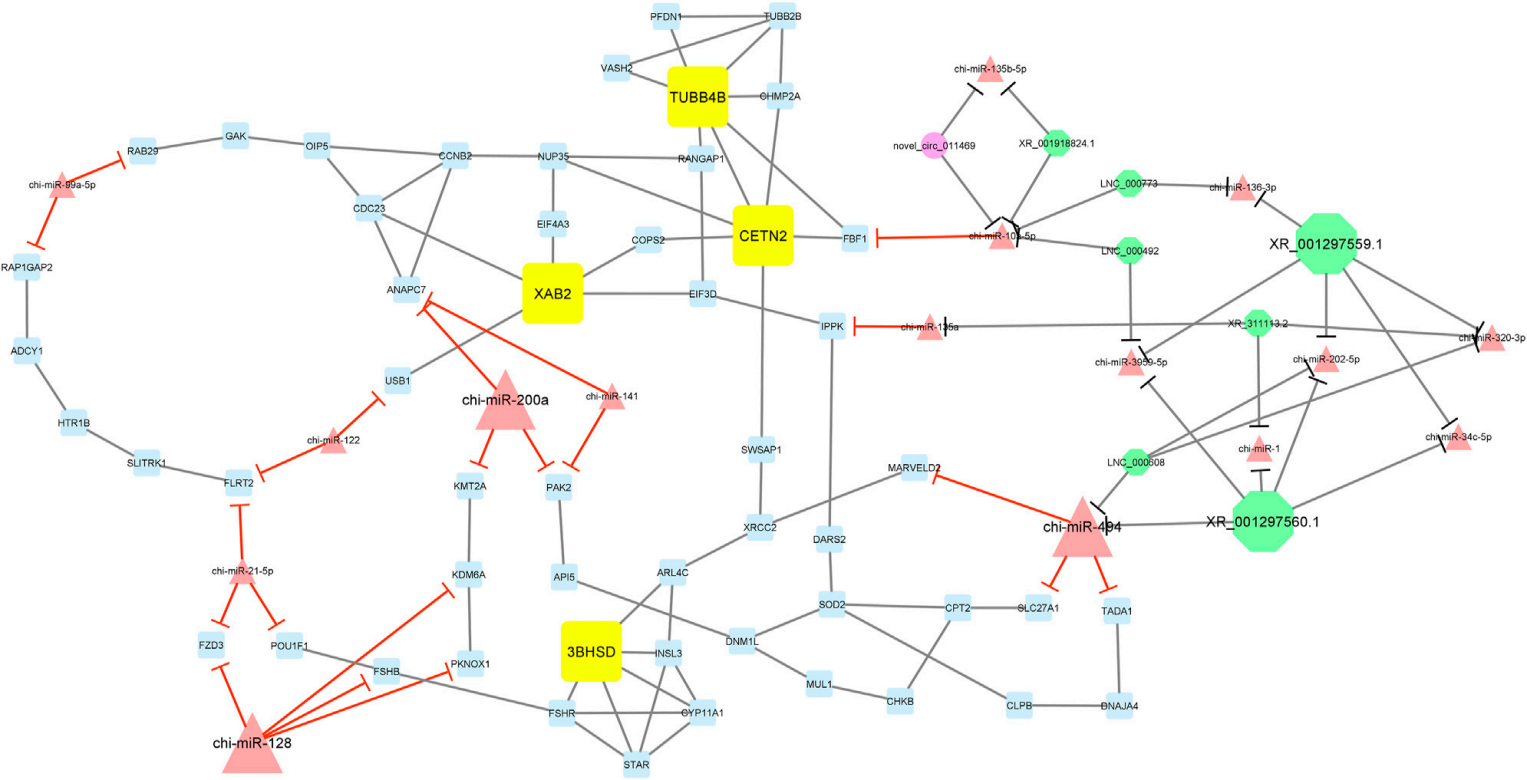
Module 3: 1 circRNA, 7 lncRNAs, 16 miRNAs, and 53 mRNAs in an interacted network were identified. In this network, circular nodes represent circRNAs, octagonal nodes represent lncRNAs, triangle nodes represent miRNAs, and quadrilateral nodes represent mRNAs/genes. Yellow quadrilateral, big triangle, and big octagonal nodes represent hub mRNAs/genes, miRNAs, and lncRNAs involved in the network, respectively. Edges indicate interactions; black edges represent mRNA–mRNA, lncRNA–miRNA, and circRNA–miRNA interactions, and red edges represent miRNA–mRNA interactions.

Furthermore, module 3 consisted of 77 nodes and 112 edges and, in detail, comprised 1 circRNA, 7 lncRNAs, 16 miRNAs, and 53 mRNAs. In this module, *TUBB4B*, *CETN2*, *XAB2*, and *3BHSD* had the most interaction with other module genes as hub genes. In addition, chi-miR-200a, chi-miR-494, and chi-miR-128, as hub miRNAs, suppressed genes *ANAPC7*, *KMT2A*, *PAK2*, *MARVELD2*, *SLC27A1*, *TADA1*, *FZD3*, *KDM6A*, *FSHB*, and *PKNOX1*. Among these, chi-miR-128 miRNA suppressed most of the genes involved in the network. Moreover, in this module, XR_001297559.1 and XR_001297560.1 circRNAs interacted with most miRNAs such as chi-miR-494, chi-miR-3959-5p, chi-miR-494, chi-miR-1, chi-miR-202-5p, chi-miR-34c-5p, chi-miR-320-3p, and chi-miR-136-3p (Figure 7).

**FIGURE 8:**
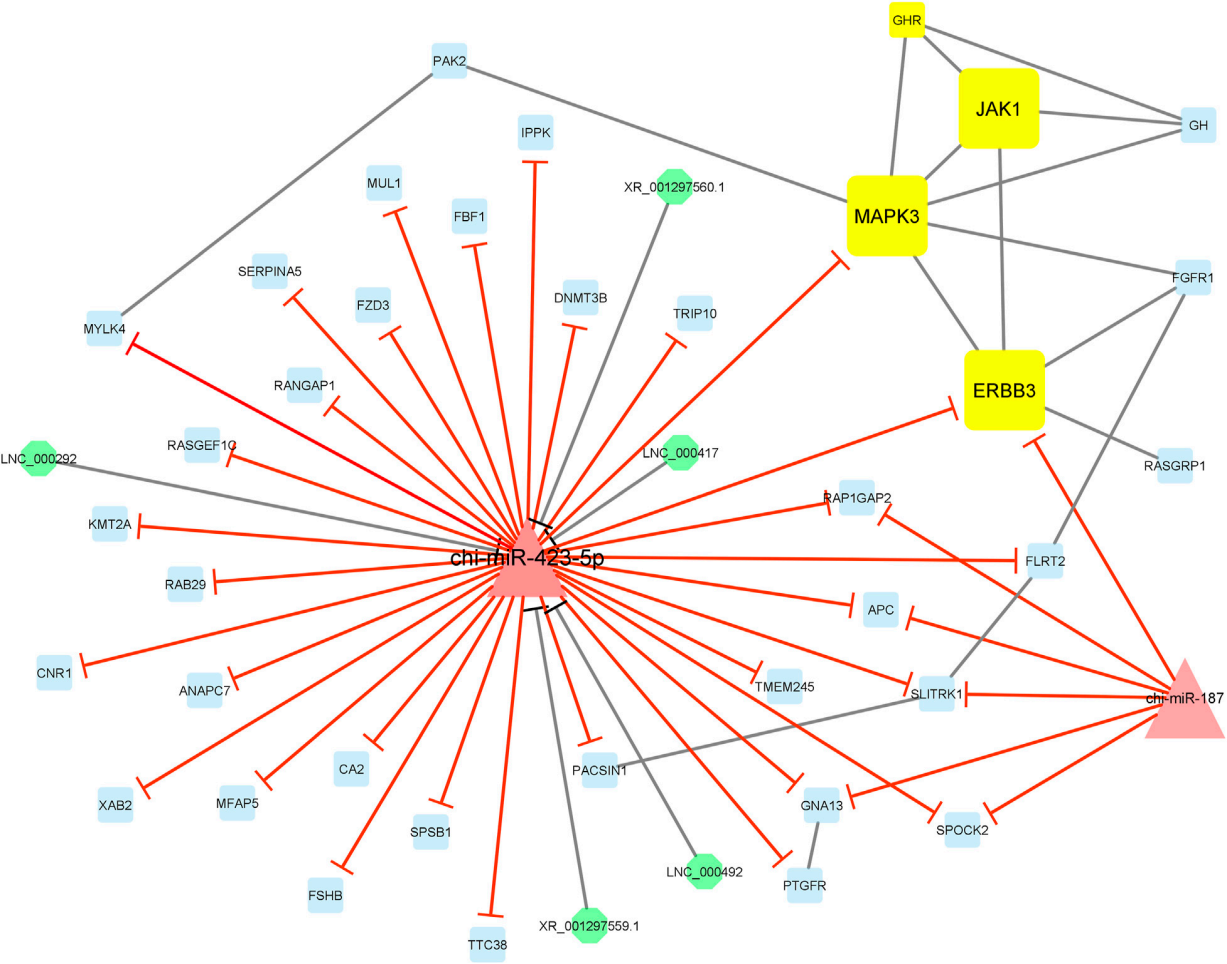
Module 4: 5 lncRNAs, 2 miRNAs, and 37 mRNAs in an interacted network were identified. In this network, octagonal nodes represent lncRNAs, triangle nodes represent miRNAs, and quadrilateral nodes represent mRNAs/genes. Yellow quadrilateral and big triangle nodes represent hub mRNAs/genes and miRNAs involved in the network, respectively. Edges indicate interactions; black edges represent mRNA–mRNA and lncRNA–miRNA interactions, and red edges represent miRNA–mRNA interactions.

Finally, module 4 consisted of 44 nodes and 59 edges and, in detail, comprised 5 lncRNAs, 2 miRNAs, and 37 mRNAs. In this module, *MAPK3*, *JAK1*, and *ERBB3* had the most interaction with other module genes as hub genes. In addition, almost all genes involved in this module were suppressed by chi-miR-423-5p and chi-miR-187 miRNAs. Moreover, chi-miR-423-5p miRNA interacted with all of the five lncRNAs, i.e., XR_001297560.1, XR_001297559.1, LNC_000292, LNC_000417, and LNC_000492, involved in the module (Figure 8).

Based on the literature, genes related to reproductive function in dairy goats, including *CCNB2*, *AR*, *ADCY1*, *DNMT3B*, *SMAD2*, *AMHR2*, *ERBB2*, and *FGFR1* genes, were specifically selected in goats with high fertility (Lai et al., 2016; Zonaed Siddiki et al., 2020). Therefore, our results provided further evidence of the association between nine hub genes (*AR*, *SMAD1*, *SMAD2*, *SMAD3*, *SMAD4*, *AMHR2*, *TGFBR2*, *CTNNB1*, and *ERBB2*) and female reproductive functions in goats. It was reported that the *ERBB2* (Erb-b2 receptor tyrosine kinase 2) gene is a steroid hormone receptor involved in calcium signaling (Zwick et al., 1999). Similarly, the *AR* (androgen receptor) gene is a protein that involves a DNA-binding transcription factor and chromatin binding; furthermore, it plays a key role in reproduction by transmitting androgen signals (Zonaed Siddiki et al., 2020). *TGFBR2* (transforming growth factor beta receptor 2) is a protein-coding gene that plays a major role in TGF-beta receptor signaling, activating SMADs, transferase activity, transferring phosphorus-containing groups, and protein tyrosine kinase activity (Yao et al., 2022). *CTNNB1* (catenin beta 1) and *TGF-β2* genes were associated with transforming growth factor beta (TGF-β), signaling pathways regulating pluripotency of stem cells, HTLV-I infection, neuroactive ligand–receptor interaction, Wnt, and Hippo signaling pathways (Li et al., 2021b). The *SMAD2*, *SMAD3*, and *SMAD4* genes play critical roles in growth and differentiation of ovarian cells, consistent with some aspects of ovulation (Li et al., 2008; Fortin et al., 2014).

Notably, the *AMHR2* (anti-Mullerian hormone receptor type 2) gene is associated with transferase activity, transport of phosphorus-containing groups, and protein tyrosine kinase activity. This gene is also involved in growth and development of ovarian follicles in cattle and goats (Monniaux et al., 2011). Furthermore, genes *BMP15* and *GDF9*, as candidate genes, are members of the beta-growth factor (TGF-β) family, directly related to twinning, increasing ovulation, and growth and development of ovarian follicles in sheep and goats (Pramod et al., 2013). The *FOXL2* gene is involved in ovarian growth and function, as well as early stages of mammalian ovarian growth (Baron et al., 2005). *FSHB* (follicle-stimulating hormone subunit beta) is a critical gene in follicle-stimulating hormone activity and peptide hormone metabolism. Moreover, variations in this gene may affect signaling of follicle differentiation and ovulation (Zi et al., 2020). The *GH* (growth hormone) gene is directly involved in nutrition-induced changes in the control of reproductive functions, e.g., ovarian follicular growth and development, cell division, and ovulation (Zhang et al., 2011). The *PRLR* (prolactin receptor) gene has been detected in various tissues, including the brain, ovary, placenta, and uterus in various mammals, especially small ruminants. This hormone is involved in many endocrine activities and is essential for reproductive function (Ozmen et al., 2011). Therefore, according to the functions of hub genes identified in the circRNA–lincRNA–lncRNA–miRNA–mRNA ceRNA regulatory network and modules, we concluded that these genes play important roles in reproductive performance and fertility of goats. In this regard, they are involved in endocrine glands, growth, cell differentiation, as well as follicle maturation, and increased ovulation and could be selected in breeding programs to increase economic benefits. In addition, most genes involved in the ceRNA regulatory network encode signaling pathways regulating pluripotency of stem cells, cytokine–cytokine receptor interaction, ovarian steroidogenesis, and neuroactive ligand–receptor interaction, thereby confirming functions of the involved genes, especially hub genes.

Signaling and metabolic pathways encoded by the genes involved in the circRNA–lincRNA–lncRNA–miRNA–mRNA ceRNA regulatory network and modules are presented in Figures 2, 3. The signaling pathways of TGF-beta, regulating pluripotency of stem cells, Hippo, MAPK, PI3K-Akt, and FoxO are encoded by the ceRNA regulatory network and modules. The TGF-beta signaling pathway encodes a large group of related structural proteins, including bone morphogenetic proteins, growth factor, and differentiation (Liu et al., 2018). Signaling pathways regulating pluripotency of stem cells are encoded by pluripotent stem cells (PSCs), which show potential to produce all three germ cell layers. It is noteworthy that embryonic stem cells (ESCs) are derived from the inner cell mass (ICM) of blastocyst-stage embryos (Mossahebi-Mohammadi et al., 2020). The Hippo signaling pathway is involved in inhibiting cell proliferation and enhancing apoptosis (Saucedo and Edgar, 2007). *MAPK3* (mitogen-activated protein kinase 3) is a gene in a MAP kinase family. This gene plays a major role in the signaling cascade that regulates various cellular processes such as proliferation, differentiation, and cell cycle progression in response to a variety of extracellular signals (Miao et al., 2016a). Functional metabolic pathways such as the cytokine–cytokine receptor interaction, ovarian steroidogenesis, neuroactive ligand–receptor interaction, and growth hormone synthesis secretion and action are also encoded in these subnetworks, with important roles in reproduction and fertility.

In this study, a computational approach with a circRNA–lincRNA–lncRNA–miRNA–mRNA ceRNA regulatory network was performed using predicted and validated expression profiles of RNAs. Spatiotemporal differential expression in various tissues, especially ovarian follicles, covered the potential roles of types of RNAs in the transcriptional and post-transcriptional regulation of genes involved in fertility. A common explanation for inconsistencies in our results was differences in applied molecular techniques (GWAS, Microarray, scRNA-seq, and simple relative gene expression), differences in ovarian tissue, time of sampling, and bioinformatics algorithms. Limitations to the present studies include the lack of a single, comprehensive dataset with similar environmental conditions and ovarian tissue from similar goat breeds.

In summary, we concluded that identified transcriptomic signatures are potentially important biomarkers to better understand functional pathways involved in fertility in female goats. Further efforts are needed to elucidate the specific biological functional types of RNAs in reproduction and fertility. Moreover, our findings integrated circRNAs, lincRNAs, lncRNAs, miRNAs, and mRNAs based on an integrated approach from bioinformatics analyses and literature mining to construct ceRNA regulatory networks. Although this can be considered a robust approach to detect significant insights into BPs, further research will be needed to confirm our results.

## 4 Conclusion

This study used a novel approach to combining various types of RNA as an integrated network in goat fertility. Analyses of scRNA-seq data resulted in identification of 150 DEGs in goats with high *versus* low fertility. Among them, 80 genes were upregulated and 70 were downregulated. Moreover, 81 mRNAs/genes, 58 circRNAs, 8 lincRNAs, 19 lncRNAs, and 55 miRNAs, all well-known types of regulatory RNAs, were obtained from literature mining. Using circRNA–lincRNA–lncRNA–miRNA–mRNA ceRNA, a regulatory network was constructed and these identified RNAs were mainly associated with transcriptional regulatory activities and signaling receptor-binding activities in terms of MFs, as well as reproductive functions such as ovulation cycle, ovarian follicle development, growth, and differentiation cells based on BPs. Furthermore, our results are a valuable resource to elucidate molecular networks and the functions of DEGs underlying ovarian follicular development, and they increase the understanding of the genetic basis of high- *versus* low-fertility goats.

## Data Availability

The datasets presented in this study can be found in online repositories. The names of the repository/repositories and accession number(s) can be found in the article/Supplementary Material.
