# Corrigendum: Construction of a circRNA–lincRNA–lncRNA–miRNA–mRNA ceRNA regulatory network identifies genes and pathways linked to goat fertility

**DOI:** 10.3389/fgene.2025.1422201

**Published:** 2025-02-18

**Authors:** Farzad Ghafouri, Mostafa Sadeghi, Abolfazl Bahrami, Masoumeh Naserkheil, Vahid Dehghanian Reyhan, Arash Javanmard, Seyed Reza Miraei-Ashtiani, Soheila Ghahremani, Herman W. Barkema, Rostam Abdollahi-Arpanahi, John P. Kastelic

**Affiliations:** ^1^ Department of Animal Science, College of Agriculture and Natural Resources, University of Tehran, Karaj, Iran; ^2^ Nuclear Agriculture Research School, Nuclear Science and Technology Research Institute, Karaj, Iran; ^3^ Animal Breeding and Genetics Division, National Institute of Animal Science, Cheonan-si, Republic of Korea; ^4^ Department of Animal Sciences, Faculty of Agriculture, University of Tabriz, Tabriz, Iran; ^5^ Department of Animal Science, Faculty of Agriculture, University of Tarbiat Modares, Tehran, Iran; ^6^ Faculty of Veterinary Medicine, University of Calgary, Calgary, AB, Canada

**Keywords:** fertility, ceRNA regulatory network, ovarian granulosa cells, goat, scRNA-seq analysis

In the published article, there was an error in **Affiliation 2**. Instead of “Biomedical Center for Systems Biology Science Munich, Ludwig-Maximilians-University, Munich, Germany,” it should be “Nuclear Agriculture Research School, Nuclear Science and Technology Research Institute, Karaj, Iran”.

The authors apologize for this error and state that this does not change the scientific conclusions of the article in any way. The original article has been updated.

