# Supplementary material for: Construction of a circRNA– lincRNA–lncRNA–miRNA–mRNA ceRNA regulatory network identifies genes and pathways linked to goat fertility
Source: Front Genet. 2023 Jul 21;14:1195480. doi: 10.3389/fgene.2023.1195480 (PMC10400778; doi:10.3389/fgene.2023.1195480)
Supplement: Supplementary file 1 [file Table1.DOCX]

**Supplementary Table S1.** Information about differentially expressed genes from granulosa cell tissue involved in goat reproductive function.

| **Gene** | **Annotation** | **Locus** | **Log2 (FC)** | **Down/Up Regulation**  **(logFC)** | **p-value** | **FDR** |
| --- | --- | --- | --- | --- | --- | --- |
| GPD1 | Glycerol-3-phosphate dehydrogenase [NAD(+)] | 5:29442866-29447843 | -9.19331 | Down | 0.0008 | 0.0047 |
| IBA57 | Iron-sulfur cluster assembly factor IBA57 | 7:105853733-105863226 | -8.55738 | Down | 0.0002 | 0.0014 |
| SEMA3B | Semaphorin 3B | 22:50165418-50173997 | -8.49522 | Down | 0.0015 | 0.0082 |
| TUBB4B | Tubulin beta chain | 11:106012194-106018981 | -8.49157 | Down | 0.0089 | 0.0355 |
| LRRN4CL | LRRN4 C-terminal like | 29:41345445-41346147 | -8.18056 | Down | 5.00E-05 | 0.0004 |
| SLC39A1 | Solute Carrier Family 39 Member 1 | 3:103673825-103676095 | -8.08753 | Down | 5.00E-05 | 0.0004 |
| SLC27A1 | Solute carrier family 27 member 1 | 7:103143596-103180185 | -7.80127 | Down | 5.00E-05 | 0.0004 |
| NENF | Neudesin neurotrophic factor | 16:69778079-69787070 | -7.70265 | Down | 0.0009 | 0.0052 |
| SSTR1 | Somatostatin receptor 1 | 21:47760560-47761733 | -7.50318 | Down | 5.00E-05 | 0.0004 |
| DPCD | Deleted in primary ciliary dyskinesia homolog | 26:29308976-29331180 | -7.42476 | Down | 5.00E-05 | 0.0004 |
| SNORA71 | Small Nucleolar RNA, H/ACA Box71 | 13:66920475-66920610 | -7.39663 | Down | 0.0001 | 0.0008 |
| ARHGEF11 | Rho guanine nucleotide exchange factor 11 | 3:106187556-106238701 | -7.37362 | Down | 5.00E-05 | 0.0004 |
| SHKBP1 | SH3KBP1 binding protein 1 | 18:50775235-50787472 | -7.31352 | Down | 5.00E-05 | 0.0004 |
| RANGAP1 | Ran GTPase activating protein 1 | 5:111333455-111356334 | -7.25519 | Down | 0.004 | 0.0180 |
| CRELD2 | Cysteine rich with EGF like domains2 | 1:4040-12024 | -7.1257 | Down | 5.00E-05 | 0.0004 |
| HSP70.1 | Heat Shock Protein 70 | 23:22440674-22442600 | -6.90368 | Down | 0.0014 | 0.0078 |
| INAVA | Innate immunity activator | 16:78051398-78071532 | -6.88814 | Down | 0.0073 | 0.0301 |
| VKORC1 | Vitamin K epoxide reductase complex subunit 1 | 25:27072303-27074270 | -6.85778 | Down | 5.00E-05 | 0.0004 |
| RASGEF1C | RasGEF Domain Family Member 1C | 7:107671877-107718519 | -6.72713 | Down | 5.00E-05 | 0.0004 |
| CETN2 | Centrin 2 | 1:168431-171062 | -6.68624 | Down | 5.00E-05 | 0.0004 |
| OR2G3 | Olfactory receptor | 7:69585165-69586107 | -6.50794 | Down | 5.00E-05 | 0.0004 |
| CEP68 | Centrosomal protein 68 | 11:63185701-63197357 | -6.50383 | Down | 5.00E-05 | 0.0004 |
| SEPTIN9 | Septin 9 | 19:53704071-53886331 | -6.4803 | Down | 0.0017 | 0.0089 |
| UPK1B | Tetraspanin | 1:63692186-63720974 | -6.3862 | Down | 0.0008 | 0.0049 |
| OR6N1 | Olfactory receptor | 3:108957408-108958347 | -6.3805 | Down | 5.00E-05 | 0.0004 |
| RBCK1 | RANBP2-type and C3HC4-type zinc finger containing 1 | 13:59919485-59936560 | -6.34288 | Down | 0.0001 | 0.0008 |
| PSENEN | Presenilin enhancer, gamma-secretase subunit | 18:47304446-47305354 | -6.25986 | Down | 5.00E-05 | 0.0004 |
| DNAJA4 | DnaJ heat shock protein family (Hsp40) member A4 | 21:30261298-30276167 | -6.21684 | Down | 5.00E-05 | 0.0004 |
| GAK | Cyclin G associated kinase | 1:38069-87617 | -6.1762 | Down | 0.0018 | 0.0096 |
| SSBP2 | Single Stranded DNA Binding Protein 2 | 7:28868886-29174470 | -6.12691 | Down | 0.0109 | 0.0419 |
| PKNOX1 | PBX/knotted 1 homeobox 1 | 1:142763190-142786107 | -6.0568 | Down | 5.00E-05 | 0.0004 |
| IPPK | Inositol-pentakisphosphate 2-kinase | 8:83954044-84011689 | -6.0167 | Down | 0.0003 | 0.0021 |
| GPANK1 | G-patch domain and ankyrin repeats 1 | 23:22327552-22329168 | -5.85729 | Down | 5.00E-05 | 0.0004 |
| PACSIN1 | Protein kinase C and casein kinase substrate in neurons 1 | 23:40113764-40120427 | -5.8411 | Down | 0.0041 | 0.01825 |
| FBRSL1 | Fibrosin like 1 | 17:26509964-26592362 | -5.68624 | Down | 0.0007 | 0.0042 |
| IZUMO4 | IZUMO family member 4 | 7:89122028-89124872 | -5.66666 | Down | 0.0007 | 0.0044 |
| SPSB1 | SplA/ryanodine receptor domain and SOCS box containing 1 | 16:42604044-42613588 | -5.64162 | Down | 5.00E-05 | 0.0004 |
| WDR13 | WD repeat domain 13 | 1:134116-140296 | -5.59433 | Down | 5.00E-05 | 0.0004 |
| PDILT | Protein disulfide isomerase like | 25:17858635-17905751 | -5.56988 | Down | 0.0023 | 0.0116 |
| MYOZ1 | Myozenin 1 | 28:16015227-16021257 | -5.51919 | Down | 0.0008 | 0.0049 |
| NUP35 | Nucleoporin NUP53 | 2:122899683-122932467 | -5.48871 | Down | 0.0130 | 0.0483 |
| CHRM5 | Cholinergic Receptor Muscarinic 5 | 10:74512949-74514548 | -5.45779 | Down | 5.00E-05 | 0.0004 |
| COG5 | Component of oligomeric golgi complex 5 | 4:71696008-72010103 | -5.45008 | Down | 0.0018 | 0.0093 |
| ADGRA1 | Adhesion G protein-coupled receptor A1 | 26:507484-543870 | -5.44769 | Down | 5.00E-05 | 0.0004 |
| SRSF11 | Serine and arginine rich splicing factor 11 | 3:45946252-45980131 | -5.44131 | Down | 0.0032 | 0.0151 |
| CHMP2A | Charged multivesicular body protein 2A | 18:67197928-67199716 | -5.4393 | Down | 5.00E-05 | 0.0004 |
| SWSAP1 | SWIM-type zinc finger 7 associated protein 1 | 7:95634673-95635959 | -5.4374 | Down | 5.00E-05 | 0.0004 |
| DMAC2L | Distal membrane arm assembly complex 2 like | 10:59821909-59825751 | -5.42204 | Down | 0.0002 | 0.0017 |
| NFKB2 | Nuclear factor kappa B subunit 2 | 26:28653518-28660053 | -5.40486 | Down | 5.00E-05 | 0.0004 |
| GLT8D1 | Glycosyltransferase 8 domain containing 1 | 22:48072305-48078217 | -5.34941 | Down | 0.0011 | 0.0064 |
| SYCP3 | Synaptonemal complex protein 3 | 5:64190769-64196613 | -5.32587 | Down | 0.0083 | 0.0336 |
| SLC39A3 | Solute Carrier Family 39 Member 3 | 7:89623705-89630642 | -5.31865 | Down | 5.00E-05 | 0.0004 |
| ADTRP | Androgen dependent TFPI regulating protein | 23:5673003-5740991 | -5.30848 | Down | 0.0061 | 0.0256 |
| SULF2 | Sulfatase 2 | 13:75686522-75810256 | -5.28739 | Down | 0.0036 | 0.0167 |
| TMEM30B | Transmembrane Protein 30B | 10:29785077-29786139 | -5.28565 | Down | 0.0005 | 0.0032 |
| ZBTB12 | Zinc finger and BTB domain containing 12 | 23:22510397-22511777 | -5.27534 | Down | 5.00E-05 | 0.0004 |
| KRT79 | Keratin 79 | 5:26826465-26836646 | -5.25043 | Down | 5.00E-05 | 0.0004 |
| VASH2 | Vasohibin 2 | 16:69248017-69285440 | -5.24606 | Down | 5.00E-05 | 0.0004 |
| TMEM245 | Transmembrane protein 245 | 8:98382231-98447074 | -5.244 | Down | 0.0048 | 0.0209 |
| CBLN1 | Cerebellin 1 precursor | 18:19573117-19576751 | -5.24325 | Down | 5.00E-05 | 0.0004 |
| TADA1 | Transcriptional adaptor 1 | 3:118046380-118060715 | -5.22978 | Down | 0.0011 | 0.0064 |
| ACYP1 | Acylphosphatase | 10:16814574-16819602 | -5.21001 | Down | 0.0131 | 0.0488 |
| SGSM2 | Small G protein signaling modulator 2 | 19:23102419-23138526 | -5.19107 | Down | 5.00E-05 | 0.0004 |
| CNR1 | Cannabinoid receptor 1 | 9:49082847-49084266 | -5.16919 | Down | 5.00E-05 | 0.0004 |
| HOXA5 | Homeobox A5 | 4:51310698-51312852 | -5.13653 | Down | 5.00E-05 | 0.0004 |
| TSSK4 | Serine/Threonine-Protein Kinase 22E | 10:80477962-80480356 | -5.11384 | Down | 0.0061 | 0.0259 |
| USP5 | Ubiquitin carboxyl-terminal hydrolase | 5:102504312-102521587 | -5.10652 | Down | 0.0126 | 0.0473 |
| MLLT11 | MLLT11 transcription factor 7 cofactor | 3:100441876-100442149 | -5.04262 | Down | 0.0109 | 0.0419 |
| MGP | Matrix Gla protein | 5:93623839-93627098 | -5.04127 | Down | 5.00E-05 | 0.0004 |
| OR5AU1 | Olfactory receptor | 10:76970180-76974342 | -5.0239 | Down | 0.0054 | 0.0233 |
| STX11 | Syntaxin 11 | 9:68625415-68626279 | 5.00549 | Up | 0.0002 | 0.0017 |
| SOD2 | Superoxide dismutase | 9:83261627-83271298 | 5.01214 | Up | 0.0129 | 0.0481 |
| PFDN1 | Prefoldin subunit 1 | 7:58990843-59062428 | 5.02657 | Up | 0.0006 | 0.0037 |
| CDC23 | Cell division cycle 23 | 7:60856747-60876211 | 5.08749 | Up | 5.00E-05 | 0.0004 |
| STX8 | Syntaxin 8 | 19:28482902-28592101 | 5.09642 | Up | 0.01 | 0.0391 |
| ZNF132 | Zinc finger protein 132 | 18:67121763-67126328 | 5.10174 | Up | 0.0009 | 0.0054 |
| LIPI | Lipase I | 1:21568829-21647958 | 5.10635 | Up | 5.00E-05 | 0.0004 |
| PCDHA10 | Protocadherin alpha 10 | 7:58411650-58586082 | 5.12512 | Up | 0.008 | 0.0325 |
| TMEM207 | Transmembrane protein 207 | 1:76570771-76591527 | 5.16965 | Up | 5.00E-05 | 0.0004 |
| XRCC2 | X-ray repair cross complementing 2 | 4:4815022-4837345 | 5.18262 | Up | 5.00E-05 | 0.0004 |
| CNBP | CCHC-type zinc finger nucleic acid binding protein | 22:59013769-59015107 | 5.18366 | Up | 0.0085 | 0.0341 |
| RASGRP1 | RAS guanyl releasing protein 1 | 10:68872595-68947433 | 5.1857 | Up | 5.00E-05 | 0.0004 |
| ETFRF1 | Electron transfer flavoprotein regulatory factor 1 | 5:83413271-83413621 | 5.26503 | Up | 0.0089 | 0.0357 |
| JAK1 | Janus Kinase 1 | 3:40348156-40481038 | 5.27441 | Up | 5.00E-05 | 0.0004 |
| ZNF800 | Zinc finger protein 800 | 4:28477140-28498277 | 5.27572 | Up | 5.00E-05 | 0.0004 |
| CHKB | Choline kinase beta | 5:118848073-118851129 | 5.32684 | Up | 5.00E-05 | 0.0004 |
| DUSP11 | Dual specificity phosphatase 11 | 11:10711079-10725548 | 5.33306 | Up | 0.0047 | 0.0205 |
| SPDEF | SAM pointed domain containing ETS transcription factor | 23:40100597-40107766 | 5.34397 | Up | 0.0134 | 0.0497 |
| CCDC63 | Coiled-coil domain containing 63 | 17:16524562-16559322 | 5.37608 | Up | 0.0001 | 0.0008 |
| DNM1L | Dynamin 1 like | 5:75702962-75764565 | 5.37919 | Up | 5.00E-05 | 0.0004 |
| CHP2 | Calcineurin like EF-hand protein 2 | 25:21348142-21353236 | 5.39604 | Up | 5.00E-05 | 0.0004 |
| XAB2 | XPA binding protein 2 | 7:96331477-96340178 | 5.40331 | Up | 5.00E-05 | 0.0004 |
| AP1AR | Adaptor related protein complex 1 associated regulatory protein | 6:13596767-13624354 | 5.40452 | Up | 0.0095 | 0.0376 |
| ERBB3 | Erb-b2 receptor tyrosine kinase 3 | 5:56593134-56613025 | 5.406 | Up | 5.00E-05 | 0.0004 |
| SYNGR2 | Synaptogyrin | 19:53194764-53197707 | 5.41746 | Up | 0.0112 | 0.0431 |
| RHOB | Ras Homolog Family Member B | 11:78108327-78108918 | 5.45498 | Up | 0.0001 | 0.0008 |
| CREBL2 | cAMP responsive element binding protein like 2 | 5:95818653-95821397 | 5.52819 | Up | 0.0116 | 0.0441 |
| CCDC117 | Coiled-coil domain containing 117 | 17:3052095-3060141 | 5.54585 | Up | 0.0006 | 0.0041 |
| EIF3D | Eukaryotic translation initiation factor 3 subunit D | 5:73623132-73635109 | 5.5514 | Up | 5.00E-05 | 0.0004 |
| COPS2 | COP9 signalosome subunit 2 | 10:42049555-42077082 | 5.5896 | Up | 5.00E-05 | 0.0004 |
| PROX2 | Prospero homeobox 2 | 10:17004319-17012979 | 5.60123 | Up | 5.00E-05 | 0.0004 |
| POU3F4 | POU domain protein | 1:6228321-6229413 | 5.60265 | Up | 0.006 | 0.0254 |
| AGR3 | Anterior gradient 3 | 4:95164479-95177238 | 5.60387 | Up | 0.0109 | 0.0419 |
| NPY2R | Neuropeptide Y receptor Y2 | 17:70710510-70711665 | 5.60543 | Up | 5.00E-05 | 0.0004 |
| DPF1 | Double PHD fingers 1 | 18:48772856-48937355 | 5.62467 | Up | 0.0101 | 0.0394 |
| IGFLR1 | IGF like family receptor 1 | 18:47299051-47300983 | 5.64985 | Up | 0.0030 | 0.0142 |
| SLC16A2 | Solute carrier family 16 member 2 | 1:8197993-8333766 | 5.68073 | Up | 5.00E-05 | 0.0004 |
| TRIP10 | Thyroid hormone receptor interactor 10 | 7:92753141-92774187 | 5.69961 | Up | 0.0013 | 0.0073 |
| TUBB2B | Tubulin beta chain | 23:15184558-15188180 | 5.7331 | Up | 5.00E-05 | 0.0004 |
| CRISPLD2 | Cysteine rich secretory protein LCCL domain containing 2 | 18:12373814-12443773 | 5.7398 | Up | 5.00E-05 | 0.0004 |
| MITD1 | Microtubule interacting and trafficking domain containing 1 | 11:4238665-4255872 | 5.75326 | Up | 0.0037 | 0.0171 |
| SLITRK1 | SLIT and NTRK like family member1 | 12:27292734-27294825 | 5.79473 | Up | 5.00E-05 | 0.0004 |
| BEND6 | BEN domain containing 6 | 23:45452279-45513291 | 5.79658 | Up | 5.00E-05 | 0.0004 |
| CAD | Carbamoyl-phosphate synthetase 2, aspartate transcarbamylase, and dihydroorotase | 11:72117108-72144088 | 5.80944 | Up | 0.0022 | 0.0112 |
| FEM1A | Fem-1 homolog A | 7:91189486-91191448 | 5.82357 | Up | 0.0125 | 0.0471 |
| RCVRN | Recoverin | 19:28760243-28768217 | 5.84603 | Up | 0.0018 | 0.0096 |
| FAM13C | Family with sequence similarity 13 member C | 28:30482369-30624879 | 5.90375 | Up | 0.0003 | 0.0021 |
| DPEP2 | Dipeptidase | 18:36724740-36729232 | 5.95426 | Up | 0.0003 | 0.00219 |
| BOLA1 | BolA family member 1 | 3:99449356-99454126 | 5.95924 | Up | 5.00E-05 | 0.0004 |
| KCNA5 | Potassium voltage-gated channel subfamily A member 5 | 5:104056739-104058536 | 5.96818 | Up | 0.0071 | 0.0295 |
| RAP1GAP2 | RAP1 GTPase activating protein 2 | 19:23547086-23647294 | 5.97446 | Up | 0.0023 | 0.0116 |
| DACT1 | Dishevelled binding antagonist of beta catenin 1 | 10:32309312-32318948 | 6.02284 | Up | 0.0005 | 0.0032 |
| MARVELD1 | MARVEL domain containing 1 | 26:32746583-32747105 | 6.03712 | Up | 5.00E-05 | 0.0004 |
| CPPED1 | Calcineurin like phosphoesterase domain containing 1 | 25:11268954-11399557 | 6.10673 | Up | 0.0003 | 0.0023 |
| ARID3B | AT-rich interaction domain 3B | 21:33529748-33577873 | 6.12061 | Up | 5.00E-05 | 0.0004 |
| MUL1 | Mitochondrial E3 ubiquitin protein ligase 1 | 2:3844040-3852316 | 6.13407 | Up | 5.00E-05 | 0.0004 |
| MARVELD2 | MARVEL domain containing 2 | 20:10328609-10344217 | 6.14671 | Up | 0.0002 | 0.0017 |
| TTC38 | Tetratricopeptide repeat domain 38 | 5:115520230-115564911 | 6.16299 | Up | 0.0014 | 0.0078 |
| TMEM150C | Transmembrane protein 150C | 6:97735724-97754568 | 6.27345 | Up | 0.0033 | 0.0154 |
| HTR1B | 5-hydroxytryptamine receptor 1B | 9:4295991-4297161 | 6.29883 | Up | 0.0004 | 0.0029 |
| ANAPC7 | Anaphase promoting complex subunit 7 | 17:16924153-16944118 | 6.34396 | Up | 0.0003 | 0.0023 |
| UBAC1 | UBA domain containing 1 | 11:102921263-102940282 | 6.34576 | Up | 0.0001 | 0.0008 |
| CA2 | Carbonic anhydrase 2 | 14:5153037-5170730 | 6.3592 | Up | 0.0002 | 0.0014 |
| PLCXD2 | Phosphatidylinositol specific phospholipase C X domain containing 2 | 1:56201170-56253248 | 6.42277 | Up | 0.0008 | 0.0049 |
| U6 | U6 snRNA phosphodiesterase | 20:36489138-36489245 | 6.57162 | Up | 5.00E-05 | 0.0004 |
| TBC1D17 | TBC1 domain family member 17 | 18:57234917-57243521 | 6.70106 | Up | 0.0001 | 0.0008 |
| SOX12 | Transcription factor SOX | 13:60010610-60011555 | 6.74636 | Up | 0.0009 | 0.0052 |
| DARS2 | Aspartyl-tRNA synthetase 2, mitochondrial | 16:53725863-53752663 | 6.74871 | Up | 5.00E-05 | 0.0004 |
| ZNF438 | Zinc finger protein 438 | 13:33660126-33789284 | 6.9421 | Up | 0.0003 | 0.0023 |
| HNRNPA0 | Heterogeneous nuclear ribonucleoprotein A0 | 7:61313482-61314397 | 6.97501 | Up | 0.0011 | 0.0059 |
| MYLK4 | Myosin light chain kinase family member 4 | 23:1429887-1497117 | 7.01171 | Up | 0.0012 | 0.0066 |
| EIF4A3 | Eukaryotic translation initiation factor 4A3 | 19:51685442-51696989 | 7.38818 | Up | 5.00E-05 | 0.0004 |
| RNASEH2B | Ribonuclease H2 Subunit B | 12:66177356-66251781 | 7.53903 | Up | 5.00E-05 | 0.0004 |
| RAB29 | RAB29, member RAS oncogene family | 16:2909440-2914165 | 7.55573 | Up | 0.0007 | 0.0042 |
| COL1A2 | Collagen type I alpha 2 chain | 4:108820557-108856519 | 7.96948 | Up | 5.00E-05 | 0.0004 |
| EXOSC1 | Exosome component 1 | 26:32953817-32959860 | 8.18305 | Up | 5.00E-05 | 0.0004 |
| CPT2 | Carnitine palmitoyltransferase 2 | 3:27762825-27785214 | 8.29948 | Up | 0.0032 | 0.0152 |
| OR2AE1 | Olfactory receptor | 25:37381903-37382830 | 8.30898 | Up | 0.0008 | 0.0049 |
| U6 | U6 snRNA phosphodiesterase | 2:134268387-134268496 | 8.67531 | Up | 5.00E-05 | 0.0004 |
| TPGS1 | Tubulin polyglutamylase complex subunit 1 | 7:67310462-67316660 | 9.28981 | Up | 0.0054 | 0.0233 |
