# Supplementary material for: Construction of a circRNA– lincRNA–lncRNA–miRNA–mRNA ceRNA regulatory network identifies genes and pathways linked to goat fertility
Source: Front Genet. 2023 Jul 21;14:1195480. doi: 10.3389/fgene.2023.1195480 (PMC10400778; doi:10.3389/fgene.2023.1195480)
Supplement: Supplementary file 2 [file Table2.DOCX]

**Supplementary Table S2.** Summary of identified DEGs, based on literature mining, and their role in goat fertility.

| **Gene** | **Annotation** | **Reference(s)** |
| --- | --- | --- |
| ADCY1 | Adenylate cyclase 1 | (Lai et al., 2016) |
| AMHR2 | Anti-Muellerian hormone type-2 receptor | (Lai et al., 2016) |
| AR | Androgen receptor | (Lai et al., 2016) |
| BMP15 | Bone morphogenetic protein 15 | (Ahlawat et al., 2016) |
| BMP4 | Bone morphogenetic protein 4 | (Sharma et al., 2013) |
| BMPRIB | Bone morphogenetic protein receptor type 1B | (Ahlawat et al., 2016) |
| CCNB2 | Cyclin B2 | (Lai et al., 2016) |
| CDH26 | Cadherin 26 | (Lai et al., 2016) |
| CMTM2 | CKLF-like MARVEL transmembrane domain containing 2 | (Kang et al., 2019) |
| CSN1S1 | Alpha-S1-casein | (Wang et al., 2018) |
| CTNNB1 | Catenin Beta 1 | (Zhang et al. 2018a) |
| CYM | Chymosin | (Lai et al., 2016) |
| DNMT3B | DNA methyltransferase 3 beta | (Lai et al., 2016) |
| ERBB2 | Erb-B2 receptor tyrosine kinase 2 | (Lai et al., 2016) |
| ESR1 | Estrogen receptor 1 | (Mohammadabadi, 2020) |
| FGFR1 | Fibroblast growth factor receptor 1 | (Lai et al., 2016) |
| FOXL2 | Forkhead Box L2 | (Sarghale et al., 2013) |
| FSHB | Follicle stimulating hormone subunit beta | (An et al., 2010) |
| FSHR | Follicle-stimulating hormone receptor | (Li et al., 2010) |
| GH | Somatotropin | (Zhang et al., 2011) |
| GHR | Growth hormone receptor | (Yang et al., 2017) |
| KDM6A | Lysine (K)-specific demethylase 6A | (Lai et al., 2016) |
| MTNR1A | Melatonin receptor type 1A | (Sarghale et al., 2013) |
| POU1F1 | POU class 1 homeobox 1; Growth hormone factor 1 | (Zhang et al., 2019) |
| POU3F4 | POU domain protein; POU class 3 homeobox 4 | (Lai et al., 2016) |
| PRLR | Prolactin receptor | (An et al., 2015) |
| PSEN2 | Presenilin 2 | (Zhang et al., 2018b) |
| SETDB2 | SET domain bifurcated histone lysine methyltransferase 2 | (Lai et al., 2016) |
| SMAD2 | SMAD family member 2 | (Lai et al., 2016) |
| SPEF2 | Sperm flagellar 2 | (Chen et al., 2019) |
| STK35 | Serine/Threonine kinase 35 | (Zhang et al., 2018b) |
| THBS1 | Thrombospondin 1 | (Miao et al., 2016) |
| SMAD4 | SMAD family member 4 | (Miao et al., 2016) |
| MAPK3 | Mitogen-activated protein kinase 3 | (Miao et al., 2016) |
| TGFB1 | Transforming growth factor beta 1 | (Miao et al., 2016) |
| SMAD3 | SMAD family member 3 | (Miao et al., 2016) |
| SMAD1 | SMAD family member 1 | (Miao et al., 2016) |
| BMP7 | Bone morphogenetic protein 7 | (Miao et al., 2016) |
| ACVR1 | Activin A receptor type 1 | (Miao et al., 2016) |
| CHRD | Chordin | (Miao et al., 2016) |
| BMPR2 | Bone morphogenetic protein receptor type 2 | (Miao et al., 2016) |
| STAR | Steroidogenic acute regulatory protein | (Tao et al., 2018) |
| FZD6 | Frizzled class receptor 6 | (Zi et al., 2017) |
| ZEB1 | Zinc finger E-box Binding homeobox 1 | (Zi et al., 2017) |
| TIMP1 | TIMP Metallopeptidase inhibitor 1 | (An et al., 2021) |
| 3BHSD | 3-beta-hydroxysteroid dehydrogenase | (An et al., 2021) |
| PTGIS | Prostaglandin I2 synthase | (An et al., 2021) |
| MMP9 | Matrix metallo peptidase 9 | (An et al., 2021) |
| PAK2 | P21 (RAC1) activated kinase 2 | (Xu et al., 2021) |
| DGUOK | Deoxyguanosine kinase | (Xu et al., 2021) |
| KMT2A | Lysine methyl transferase 2A | (Xu et al., 2021) |
| APC | APC Regulator of WNT signaling pathway | (Xu et al., 2021) |
| CLPB | Caseinolytic mitochondrial matrix peptidase chaperone subunit B | (Xu et al., 2021) |
| SUDS3 | SDS3 Homolog, SIN3A corepressor complex component | (Xu et al., 2021) |
| FBF1 | Fas Binding Factor 1 | (Xu et al., 2021) |
| NUFIP2 | Nuclear FMR1 interacting protein 2 | (Xu et al., 2021) |
| API5 | Apoptosis inhibitor 5 | (Li et al., 2021) |
| KRR1 | KRR1 Small subunit processome component homolog | (Li et al., 2021) |
| NOP56 | NOP56 Ribonucleoprotein | (Li et al., 2021) |
| WNT5A | Wnt family member 5A | (Li et al., 2021) |
| OIP5 | Opa interacting protein 5 | (Li et al., 2021) |
| FLRT2 | Fibronectin leucine rich transmembrane protein 2 | (Li et al., 2021) |
| GNA13 | G Protein subunit alpha 13 | (Li et al., 2021) |
| TGFB2 | Transforming growth factor beta 2 | (Li et al., 2021) |
| TGFBR2 | Transforming growth factor beta receptor 2 | (Li et al., 2021) |
| BUB1 | BUB1 Mitotic checkpoint serine/threonine kinase | (Ling et al., 2017) |
| MOS | MOS Proto-oncogene, serine/threonine kinase | (Ling et al., 2017) |
| PIWIL3 | Piwi like RNA-mediated gene silencing 3 | (Zhao et al. 2020) |
| ACTR3 | Actin related protein 3 | (Zhao et al., 2020) |
| TNFAIP6 | TNF Alpha induced protein 6 | (Zou et al., 2020) |
| INSL3 | Insulin like 3 | (Zou et al., 2020) |
| LHCGR | Luteinizing hormone/choriogonadotropin receptor | (Zou et al., 2020) |
| ARL4C | ADP Ribosylation factor like GTPase 4C | (Zou et al., 2020) |
| CD36 | CD36 Molecule | (Zou et al., 2020) |
| CYP11A1 | Cytochrome P450 family 11 subfamily A member 1 | (Zou et al., 2020) |
| AMDHD1 | Amidohydrolase domain containing 1 | (Zou et al., 2020) |
| SPOCK2 | SPARC (Osteonectin), Cwcv and kazal like domains proteoglycan 2 | (Zou et al., 2020) |
| MFAP5 | Microfibril associated protein 5 | (Zou et al., 2020) |
| CCL21 | C-C Motif chemokine ligand 21 | (Zou et al., 2020) |
| PTGFR | Prostaglandin F receptor | (Zou et al., 2020) |
| SERPINA5 | Serpin family A member 5 | (Zou et al., 2020) |

**References**

Ahlawat, S., Sharma, R., Roy, M., Mandakmale, S., Prakash, V., and Tantia, M. (2016). “Genotyping of novel SNPs in BMPR1B, BMP15, and GDF9 genes for association with prolificacy in seven Indian goat breeds.” *Anim. Biotechnol.* 27, 199-207.

An, X., Hou, J., Gao, T., Lei, Y., Li, G., Song, Y., et al. (2015). “Single-nucleotide polymorphisms g. 151435C> T and g. 173057T> C in PRLR gene regulated by bta-miR-302a are associated with litter size in goats.” *Theriogenology*. 83, 1477-83.

An, X., Zhang, Y., Li, F., Wang, Z., Yang S., and Cao, B. (2021). “Whole transcriptome analysis: implication to estrous cycle regulation.” *Biology*, 10(464), 1-15.

An, X.P., Dan, H., Hou, J.X., Guang, L., Wang, Y.N., Ling, L., et al. (2010). “Polymorphism of exon 2 of FSHβ gene and its relationship with reproduction performance in two goat breeds.” *Agric. Sci. China.* 9, 880-6.

Chen, M., Yan, H., Wang, K., Cui, Y., Chen, R., Liu, J., et al. (2019). “Goat SPEF2: Expression profile, indel variants identification and association analysis with litter size.” *Theriogenology*. 139, 147-55.

Kang, Z., Zhang, S., He, L., Zhu, H., Wang, Z., Yan, H., et al. (2019). “A 14-bp functional deletion within the CMTM2 gene is significantly associated with litter size in goat.” *Theriogenology*. 139, 49-57.

Lai, F.N., Zhai, H.L., Cheng, M., Ma, J.Y., Cheng, S.F., Ge, W., et al. (2016). “Whole-genome scanning for the litter size trait associated genes and SNPs under selection in dairy goat (Capra hircus).” *Sci. Rep.* 6, 1-12.

Li, Y., Xu, X., Deng, M., Zou, X., Zhao, Z. Huang, S., et al. (2021). “Identification and comparative analysis of long non-coding RNAs in high- and low-fecundity goat ovaries during estrus.” *Frontiers Genetics*, 12(648158), 1-11.

Li, Y., Zhang, L., Shang, L., Wang, H., Zou, H., Zhang, H., et al. (2010). “Genetic polymorphisms at three loci of PRLR and FSHR gene correlate with litter size in Chinese Haimen goat.” *J. Anim. Vet. Adv.* 9, 2835-8.

Ling, Y., Xu, L., Zhu, L., Sui, M., Zheng, Q., Li, W., et al. (2017). “Identification and analysis of differentially expressed long non-coding RNAs between multiparous and uniparous goat (*Capra hircus*) ovaries.” *Plos One,* 12(9), 1-16.

Miao, X., Luo, Q., Zhao, H., and Qin, X. (2016). “Genome-wide analysis of miRNAs in the ovaries of Jining Grey and Laiwu Black goats to explore the regulation of fecundity.” *Scientific Reports,* 6(1), 1-9.

Mohammadabadi, M. (2020). “Expression of ESR1 gene in Raini Cashmere goat using Real Time PCR.” *Agric. Biotechnol. J.* 12, 177-92.

Sarghale, A., Shahrebabak, H., Amini, H., and Kholghi, M. (2013). “The role of major genes in main productive and economical traits in goat.” *Genetics in the Third Millennium*. 11, 3136-55.

Sharma, R., Ahlawat, S., Maitra, A., Roy, M., Mandakmale, S., and Tantia, M. (2013). “Polymorphism of BMP4 gene in Indian goat breeds differing in prolificacy.” *Gene*. 532, 140-5.

Tao, H., Xiong, Q., Zhang, F., Zhang, N., Liu, Y., Suo, X., et al. (2018). “Circular RNA profiling reveals chi_circ_0008219 function as microRNA sponges in pre-ovulatory ovarian follicles of goats (*Capra hircus*).” *Genomics,* 110, 257–266.

Wang, K., Yan, H., Xu, H., Yang, Q., Zhang, S., Pan, C., et al. (2018). “A novel indel within goat casein alpha S1 gene is significantly associated with litter size.” *Gene*. 671, 161-9.

Xu, L., Liu, C., Na, R., Zhang, W., He, Y., Yuan, Y., et al. (2021). “Genetic basis of follicle development in Dazu Black Goat by whole-transcriptome sequencing.” *Animals*, 11(3536), 1-17.

Yang, Q., Yan, H., Li, J., Xu, H., Wang, K., Zhu, H., et al. (2017). “A novel 14-bp duplicated deletion within goat GHR gene is significantly associated with growth traits and litter size.” *Anim. Genet.* 48, 499-500.

Zhang, C., Liu, Y., Huang, K., Zeng, W., Xu, D., Wen, Q., et al. (2011). “The association of two single nucleotide polymorphisms (SNPs) in growth hormone (GH) gene with litter size and superovulation response in goat-breeds.” *Genet. Mol. Biol.* 34, 49-55.

Zhang, R.Q., Lai, F.N., Wang, J.J., Zhai, H.L., Zhao, Y., Sun, Y.J., et al. (2018b). “Analysis of the SNP loci around transcription start sites related to goat fecundity trait base on whole genome resequencing.” *Gene*. 643, 1-6.

Zhang, X., Yan, H., Wang, K., Zhou, T., Chen, M., Zhu, H., et al. (2018a). “Goat CTNNB1: mRNA expression profile of alternative splicing in testis and association analysis with litter size.” *Gene*. 679, 297-304.

Zhang, Y., Cui, W., Yang, H., Wang, M., Yan, H., Zhu, H., et al. (2019). “A novel missense mutation (L280V) within POU1F1 gene strongly affects litter size and growth traits in goat.” *Theriogenology*. 135, 198-203.

Zhao, Z., Zou, X., Lu, T., Deng, M., Li, Y., Guo, Y., et al. (2020). “Identification of mRNAs and lncRNAs Involved in the regulation of follicle development in goat.” *Frontiers Genetics*, 11(589076), 1-10.

Zi, X., Lu J., and Ma, L. (2017). “Identification and comparative analysis of the ovarian microRNAs of prolific and non-prolific goats during the follicular phase using high-throughput sequencing.” *Scientific Reports,* 7(1), 1-10.

Zou, X., Lu, T., Zhao, Z., Liu, G., Lian, Z., Guo, Y., et al. (2020). “Comprehensive analysis of mRNAs and miRNAs in the ovarian follicles of uniparous and multiple goats at estrus phase.” *BMC Genomics*, 21(267), 1-15.
