# Supplementary material for: Construction of a circRNA– lincRNA–lncRNA–miRNA–mRNA ceRNA regulatory network identifies genes and pathways linked to goat fertility
Source: Front Genet. 2023 Jul 21;14:1195480. doi: 10.3389/fgene.2023.1195480 (PMC10400778; doi:10.3389/fgene.2023.1195480)
Supplement: Supplementary file 3 [file Table5.DOCX]

**Supplementary Table S5.** Summary of identified LncRNAs, based on literature mining, and their role in goat fertility.

| **LncRNAs** | **Reference(s)** |
| --- | --- |
| LNC_000029 | Tao et al., 2022 |
| LNC_000103 | Tao et al., 2022 |
| LNC_000155 | Tao et al., 2022 |
| LNC_000292 | Tao et al., 2022 |
| LNC_000417 | Tao et al., 2022 |
| LNC_000492 | Tao et al., 2022 |
| LNC_000608 | Tao et al., 2022 |
| LNC_000612 | Tao et al., 2022 |
| LNC_000773 | Tao et al., 2022 |
| XR_001295796 | An et al., 2021 |
| XR_001296481 | An et al., 2021 |
| XR_001918824.1 | Xu et al., 2021 |
| XR_001295829.1 | Tao et al., 2022 |
| XR_001297559.1 | Tao et al., 2022 |
| XR_001297560.1 | Tao et al., 2022 |
| XR_310214.2 | Tao et al., 2022 |
| XR_311113.2 | Tao et al., 2022 |
| XR_311288.2 | Tao et al., 2022 |
| XR_311364.2 | Tao et al., 2022 |

**References**

An, X., Zhang, Y., Li, F., Wang, Z., Yang S., and Cao, B. (2021). “Whole transcriptome analysis: implication to estrous cycle regulation.” *Biology*, 10(464), 1-15.

Tao, H., Yang, J., Zhang, P., Zhang, N., Suo, X., Li, X., et al. (2022). Characterization of XR_311113.2 as a microRNA sponge for pre-ovulatory ovarian follicles of goats via long noncoding RNA profile and bioinformatics analysis. Frontiers Genetics, 12(760416), 1-12.

Xu, L., Liu, C., Na, R., Zhang, W., He, Y., Yuan, Y., et al. (2021). “Genetic basis of follicle development in Dazu Black Goat by whole-transcriptome sequencing.” *Animals*, 11(3536), 1-17.
