# Supplementary material for: Construction of a circRNA– lincRNA–lncRNA–miRNA–mRNA ceRNA regulatory network identifies genes and pathways linked to goat fertility
Source: Front Genet. 2023 Jul 21;14:1195480. doi: 10.3389/fgene.2023.1195480 (PMC10400778; doi:10.3389/fgene.2023.1195480)
Supplement: Supplementary file 4 [file Table3.DOCX]

**Supplementary Table S3.** Summary of identified CircRNAs, based on literature mining, and their role in goat fertility.

| **CircRNAs** | **Reference(s)** |
| --- | --- |
| chi_circ_008176 | An et al., 2021 |
| chi_circ_008550 | An et al., 2021 |
| chi_circ_012702 | An et al., 2021 |
| chi_circ_020053 | An et al., 2021 |
| chi_circ_016569 | An et al., 2021 |
| chi_circ_014616 | An et al., 2021 |
| chi_circ_003778 | An et al., 2021 |
| chi_circ_016289 | An et al., 2021 |
| chi_circ_017163 | An et al., 2021 |
| chi_circ_015054 | An et al., 2021 |
| chi_circ_015051 | An et al., 2021 |
| chi_circ_016861 | An et al., 2021 |
| chi_circ_012430 | An et al., 2021 |
| chi_circ_005991 | An et al., 2021 |
| chi_circ_003216 | An et al., 2021 |
| chi_circ_000535 | An et al., 2021 |
| chi_circ_014158 | An et al., 2021 |
| chi_circ_013341 | An et al., 2021 |
| chi_circ_019441 | An et al., 2021 |
| chi_circ_006262 | An et al., 2021 |
| chi_circ_010355 | An et al., 2021 |
| chi_circ_007584 | An et al., 2021 |
| chi_circ_015571 | An et al., 2021 |
| chi_circ_008978 | An et al., 2021 |
| chi_circ_009965 | An et al., 2021 |
| chi_circ_002024 | An et al., 2021 |
| chi_circ_010424 | An et al., 2021 |
| chi_circ_020360 | An et al., 2021 |
| chi_circ_015412 | An et al., 2021 |
| chi_circ_007739 | An et al., 2021 |
| chi_circ_017629 | An et al., 2021 |
| chi_circ_011671 | An et al., 2021 |
| chi_circ_010070 | An et al., 2021 |
| chi_circ_010069 | An et al., 2021 |
| chi_circ_001028 | An et al., 2021 |
| chi_circ_012843 | An et al., 2021 |
| chi_circ_018150 | An et al., 2021 |
| chi_circ_003502 | An et al., 2021 |
| chi_circ_000523 | An et al., 2021 |
| chi_circ_020719 | An et al., 2021 |
| chi_circ_021133 | An et al., 2021 |
| chi_circ_001061 | An et al., 2021 |
| chi_circ_016289 | An et al., 2021 |
| chi_circ_016545 | An et al., 2021 |
| chi_circ_001270 | An et al., 2021 |
| chi_circ_017606 | An et al., 2021 |
| chi_circ_0058 | An et al., 2021 |
| chi_circ_020690 | An et al., 2021 |
| chi_circ_009251 | An et al., 2021 |
| chi_circ_006457 | An et al., 2021 |
| chi_circ_0008219 | Tao et al., 2018 |
| novel_circ_009670 | Xu et al., 2021 |
| novel_circ_006849 | Xu et al., 2021 |
| novel_circ_003318 | Xu et al., 2021 |
| novel_circ_010731 | Xu et al., 2021 |
| novel_circ_011410 | Xu et al., 2021 |
| novel_circ_011469 | Xu et al., 2021 |
| novel_circ_009670 | Xu et al., 2021 |

**References**

An, X., Zhang, Y., Li, F., Wang, Z., Yang S., and Cao, B. (2021). “Whole transcriptome analysis: implication to estrous cycle regulation.” *Biology*, 10(464), 1-15.

Tao, H., Xiong, Q., Zhang, F., Zhang, N., Liu, Y., Suo, X., et al. (2018). “Circular RNA profiling reveals chi_circ_0008219 function as microRNA sponges in pre-ovulatory ovarian follicles of goats (Capra hircus).” *Genomics,* 110, 257–266.

Xu, L., Liu, C., Na, R., Zhang, W., He, Y., Yuan, Y., et al. (2021). “Genetic basis of follicle development in Dazu Black Goat by whole-transcriptome sequencing.” *Animals*, 11(3536), 1-17.
