# Supplementary material for: Construction of a circRNA– lincRNA–lncRNA–miRNA–mRNA ceRNA regulatory network identifies genes and pathways linked to goat fertility
Source: Front Genet. 2023 Jul 21;14:1195480. doi: 10.3389/fgene.2023.1195480 (PMC10400778; doi:10.3389/fgene.2023.1195480)
Supplement: Supplementary file 5 [file Table4.DOCX]

**Supplementary Table S4.** Summary of identified LincRNAs, based on literature mining, and their role in goat fertility.

| **LincRNAs** | **Reference(s)** |
| --- | --- |
| ENSCHIT00000001883 | Li et al., 2021 |
| ENSCHIG00000000641 | Li et al., 2021 |
| ENSCHIG00000000774 | Li et al., 2021 |
| ENSCHIG00000000886 | Li et al., 2021 |
| ENSCHIG00000000609 | Li et al., 2021 |
| ENSCHIG00000002761 | Li et al., 2021 |
| ENSCHIT00000000834 | Zhao et al., 2020 |
| ENSCHIT00000009455 | Zhao et al., 2020 |

**References**

Li, Y., Xu, X., Deng, M., Zou, X., Zhao, Z. Huang, S., et al. (2021). “Identification and comparative analysis of long non-coding RNAs in high- and low-fecundity goat ovaries during estrus.” *Frontiers Genetics*, 12(648158), 1-11.

Zhao, Z., Zou, X., Lu, T., Deng, M., Li, Y., Guo, Y., et al. (2020). “Identification of mRNAs and lncRNAs Involved in the regulation of follicle development in goat.” *Frontiers Genetics*, 11(589076), 1-10.
