# Supplementary material for: Construction of a circRNA– lincRNA–lncRNA–miRNA–mRNA ceRNA regulatory network identifies genes and pathways linked to goat fertility
Source: Front Genet. 2023 Jul 21;14:1195480. doi: 10.3389/fgene.2023.1195480 (PMC10400778; doi:10.3389/fgene.2023.1195480)
Supplement: Supplementary file 6 [file Table6.DOCX]

**Supplementary Table S6.** Summary of identified miRNAs, based on literature mining, and their role in goat fertility.

| **miRNAs** | **Reference(s)** |
| --- | --- |
| chi-miR-21-3p | An et al., 2021 |
| chi-miR-202-3p | An et al., 2021 |
| chi-miR-210-x | An et al., 2021 |
| chi-miR-125a-3p | An et al., 2021 |
| chi-miR-3195-x | An et al., 2021 |
| chi-miR-9851-y | An et al., 2021 |
| chi-miR-323a-3p | An et al., 2021 |
| chi-miR-92a-5p | An et al., 2021 |
| chi-miR-9027-y | An et al., 2021 |
| chi-miR-223-3p | An et al., 2021 |
| chi-miR-29c | Ling et al., 2014 |
| chi-miR-187 | Miao et al., 2016 |
| chi-miR-874-3p | Miao et al., 2016 |
| chi-miR-145-3p | Miao et al., 2016 |
| chi-miR-183 | Miao et al., 2016 |
| chi-miR-655 | Miao et al., 2016 |
| chi-miR-9-5p | Miao et al., 2016 |
| chi-miR-34c-5p | Tao et al., 2018 |
| chi-miR-483 | Tao et al., 2018 |
| chi-miR-1468-3p | Tao et al., 2018 |
| chi-miR-125b-5p | Tao et al., 2022 |
| chi-miR-320-3p | Tao et al., 2022 |
| chi-miR-28-3p | Tao et al., 2022 |
| chi-miR-202-5p | Tao et al., 2022 |
| chi-miR-494 | Tao et al., 2022 |
| chi-miR-424-5p | Tao et al., 2022 |
| chi-miR-224-5p | Tao et al., 2022 |
| chi-miR-136-3p | Tao et al., 2022 |
| chi-miR-3958-3p | Tao et al., 2022 |
| chi-miR-3959-5p | Tao et al., 2022 |
| chi-miR-1 | Tao et al., 2022 |
| chi-miR-423-5p | Tao et al., 2022 |
| chi-miR-708-3p | Tao et al., 2022 |
| chi-miR-450-5p | Tao et al., 2022 |
| chi-miR-3955-5p | Tao et al., 2022 |
| chi-miR-135a | Tao et al., 2022 |
| chi-miR-544-5p | Tao et al., 2022 |
| chi-miR-7g-3p | Xu et al., 2021 |
| chi-miR-10a-5p | Xu et al., 2021 |
| chi-miR-1306-3p | Xu et al., 2021 |
| chi-miR-135b-5p | Xu et al., 2021 |
| chi-miR-128 | Xu et al., 2021 |
| chi-miR-2765-x | Xu et al., 2021 |
| chi-miR-376-y | Xu et al., 2021 |
| chi-miR-383 | Xu et al., 2021 |
| chi-miR-4286-z | Xu et al., 2021 |
| chi-miR-6412-y | Xu et al., 2021 |
| chi-miR-99a-5p | Zi et al., 2017 |
| chi-miR-21-5p | Zi et al., 2017 |
| chi-miR-200a | Zou et al., 2020 |
| chi-miR-141 | Zou et al., 2020 |
| chi-miR-182 | Zou et al., 2020 |
| chi-miR-206 | Zou et al., 2020 |
| chi-miR-122 | Zou et al., 2020 |
| chi-miR-133b | Zou et al., 2020 |

**References**

An, X., Zhang, Y., Li, F., Wang, Z., Yang S., and Cao, B. (2021). “Whole transcriptome analysis: implication to estrous cycle regulation.” *Biology*, 10(464), 1-15.

Ling, Y., Xu, L., Zhu, L., Sui, M., Zheng, Q., Li, W., et al. (2017). “Identification and analysis of differentially expressed long non-coding RNAs between multiparous and uniparous goat (*Capra hircus*) ovaries.” *Plos One,* 12(9), 1-16.

Miao, X., Luo, Q., Zhao, H., and Qin, X. (2016). “Genome-wide analysis of miRNAs in the ovaries of Jining Grey and Laiwu Black goats to explore the regulation of fecundity.” *Scientific Reports,* 6(1), 1-9.

Tao, H., Xiong, Q., Zhang, F., Zhang, N., Liu, Y., Suo, X., et al. (2018). “Circular RNA profiling reveals chi_circ_0008219 function as microRNA sponges in pre-ovulatory ovarian follicles of goats (*Capra hircus*).” *Genomics,* 110, 257–266.

Tao, H., Yang, J., Zhang, P., Zhang, N., Suo, X., Li, X., et al. (2022). Characterization of XR_311113.2 as a microRNA sponge for pre-ovulatory ovarian follicles of goats via long noncoding RNA profile and bioinformatics analysis*. Frontiers Genetics*, 12(760416), 1-12.

Xu, L., Liu, C., Na, R., Zhang, W., He, Y., Yuan, Y., et al. (2021). “Genetic basis of follicle development in Dazu Black Goat by whole-transcriptome sequencing.” *Animals*, 11(3536), 1-17.

Zi, X., Lu J., and Ma, L. (2017). “Identification and comparative analysis of the ovarian microRNAs of prolific and non-prolific goats during the follicular phase using high-throughput sequencing.” *Scientific Reports,* 7(1), 1-10.

Zou, X., Lu, T., Zhao, Z., Liu, G., Lian, Z., Guo, Y., et al. (2020). “Comprehensive analysis of mRNAs and miRNAs in the ovarian follicles of uniparous and multiple goats at estrus phase.” *BMC Genomics*, 21(267), 1-15.
